# Closed-Loop Integration of Neural Ambiguity, Gravity, and Line-of-Sight Estimators for GNSS/IMU/SAL Rocket GNC

**DOI:** 10.3390/s26196177

**Published:** 2026-09-29

**Authors:** Raúl de Celis, Luis Cadarso

**Affiliations:** Aerospace Systems and Transport Research Group, Transportation Research and Network Data Science Center (TRENDS), Rey Juan Carlos University, Fuenlabrada, 28942 Madrid, Spain; raul.decelis@urjc.es

**Keywords:** rocket guidance, GNSS/IMU fusion, neural networks, semi-active laser seeker, quadrant detector, terminal guidance, proportional navigation, six-degree-of-freedom simulation

## Abstract

This paper presents the closed-loop integration of three neural estimators within a physically based guidance, navigation, and control (GNC) architecture for a canard-controlled rocket. The estimators support Global Navigation Satellite System (GNSS) carrier-phase ambiguity processing, reconstruct the gravity vector in body axes, and correct the terminal line-of-sight (LOS) estimate obtained by fusing GNSS, inertial measurement unit (IMU), and semi-active laser (SAL) quadrant-detector information. Building on earlier specialized or partially integrated studies, the contribution is the simultaneous embedding of the three estimators in a common nonlinear six-degree-of-freedom closed loop and their evaluation, against a matched model-based baseline and an eight-configuration ablation study, under nominal, wind, high-angular-rate, GNSS-degradation, and combined-disturbance scenarios. By combining complementary attitude, gravity, and target-relative information while retaining physical validation and model-based fallback, the architecture is designed to improve navigation resilience and functional coverage across multiple sensor-degradation modes. All 500 closed-loop evaluation trajectories completed without numerical divergence. The overall trajectory-averaged squared vector errors were $4.13\times 10^{-3}\;m^{2}/s^{4}$ for gravity and $1.59\times 10^{-3}$(dimensionless, corresponding to a 2.3° RMS-equivalent angular error) for LOS; under combined disturbances, their mean errors were 3.08 and 2.99 times the corresponding nominal values. Relative to the matched model-based baseline, the integrated architecture improved the GNSS ambiguity fix-success rate from 96.4% to 97.9% and reduced terminal-guidance circular error probable (CEP50) from 0.49 m to 0.35 m (an absolute reduction of 0.140 m; paired-bootstrap 95% CI [0.118,0.156] m), corresponding to a 28.6% relative reduction, and the ablation study indicated a near-additive, monotonically improving contribution from each of the three modules. The results demonstrate simultaneous operation of the three modules as active components of the propagated GNC loop over the tested conditions, with a quantified improvement over the matched conventional configuration.

## 1. Introduction

Accurate terminal guidance remains one of the most demanding challenges in modern artillery rockets and tactical missile systems. Although significant advances have been achieved in propulsion technologies, aerodynamic design, onboard electronics, and actuation systems, impact accuracy is still heavily influenced by the nonlinear and highly coupled nature of rocket flight dynamics. Small perturbations introduced during launch, boost, or atmospheric flight can evolve into substantial miss distances. Thrust dispersion and the strong spin/control coupling characteristic of rotating, fin-controlled projectiles are documented for high-dynamic rotating artillery rockets [1] and for spin-stabilized fin-controlled configurations [2]. Aerodynamic and manufacturing-related uncertainties are similarly evidenced by high-fidelity, multi-fidelity vehicle-design and simulation studies [3], while structural asymmetries and control-authority limitations under co-evolutionary control design are reported in [4]. These effects become increasingly critical during the terminal phase, where the remaining flight time is short and the vehicle operates under rapidly changing aerodynamic conditions.

The increasing demand for precision-guided munitions has motivated the development of advanced Guidance, Navigation, and Control (GNC) architectures capable of reducing dispersion while maintaining low acquisition and operational costs [5]. Modern artillery systems increasingly rely on sensor and information fusion to compensate for uncertainties and improve terminal accuracy, a need documented in the command-and-control data-fusion literature [6] and, at the navigation-architecture level, in comparative INS/GPS integration studies [7] and nonlinear INS/GNSS observer designs [8]. The availability of inertial sensors, compact satellite-navigation receivers and embedded computational resources has enabled the incorporation of navigation and guidance capabilities into platforms that were traditionally considered purely ballistic systems.

Among available navigation technologies, the integration of inertial navigation systems (INS) and Global Navigation Satellite Systems (GNSS) has become the de facto standard for aerospace navigation applications. INS measurements provide high-rate acceleration and angular velocity information, ensuring continuity of navigation during temporary satellite outages [7], whereas GNSS measurements provide globally referenced position and velocity estimates with bounded long-term drift [8]. Consequently, hybrid INS/GNSS architectures have demonstrated substantial improvements in navigation performance when compared with standalone inertial or satellite-based solutions.

Nevertheless, despite their widespread adoption, INS/GNSS architectures still exhibit significant limitations in highly dynamic aerospace environments. Inertial sensors suffer from bias accumulation, scale-factor uncertainty, vibration sensitivity, and thermal drift. Similarly, GNSS measurements are affected by multipath propagation and long-correlation-time coordinate noise [9], as well as by signal attenuation and degraded satellite geometry under airframe multipath conditions [10]. These effects become particularly relevant during terminal interception, where even small navigation errors can translate into large angular deviations in the estimated line-of-sight (LOS) vector.

The LOS estimation problem is especially important in proportional-navigation-based guidance systems. Classical guidance laws compute steering commands from LOS rate measurements and closing velocity estimates, following the LOS-reconstruction formulation for faster homing guidance [11] and its robust discrete-time proportional-derivative extension [12]; a related integrated guidance/autopilot formulation based on terminal sliding-mode control is reported in [13]. Since LOS information is commonly reconstructed from relative position estimates, its accuracy depends directly on the quality of the navigation solution. As the distance to the target decreases, small position errors become geometrically amplified, potentially degrading terminal guidance performance and increasing miss distance. Consequently, improving LOS observability has become a major objective in guided rocket research.

Numerous approaches have been proposed to improve terminal observability and guidance robustness. High-dynamic rotating artillery rockets exhibit strong spin/control coupling [1]. Spin-stabilized fin-controlled projectiles impose related guidance and control constraints [2], while co-evolutionary control design [4] and independent pitch–yaw control for spinning vehicles [14] address complementary limitations in control authority. Together, these characteristics complicate navigation and control design and motivate estimation architectures capable of maintaining accuracy under highly nonlinear conditions.

One promising solution consists of incorporating complementary sensing modalities during the terminal phase. Semi-active laser (SAL) guidance systems have been widely employed in precision-guided munitions because they provide direct angular information about target location. Unlike GNSS-based LOS estimation, which is derived indirectly from relative position calculations, SAL seekers measure the reflected laser spot directly in seeker coordinates. Consequently, LOS estimation becomes less sensitive to accumulated navigation errors and inertial drift.

Among available SAL technologies, quadrant photodetectors offer an attractive solution due to their low mass, reduced computational requirements, and relatively simple hardware implementation. Configurable quadrant-detector design and position sensing are described in [15], whereas a common-aperture infrared/laser optical implementation is presented in [16]. These devices estimate target direction from the displacement of the reflected laser spot on the detector surface. However, their performance may be affected by detector nonlinearity, optical noise, atmospheric attenuation, body rotation, and calibration errors. Accurate centroid extraction therefore becomes a key element in the overall guidance architecture.

Previous research addressed several of these challenges independently. In [17], spot-centroid determination algorithms were developed for semi-active laser photodiodes used in artillery applications, providing robust LOS estimation capabilities under realistic operating conditions. Subsequently, the integration of GNSS, IMU, and laser measurements was investigated through a hybrid guidance architecture specifically designed for artillery rockets [18]. The results demonstrated that combining direct LOS measurements with conventional navigation sensors significantly improves terminal guidance accuracy.

Another major challenge in guided rocket systems concerns attitude determination. Accurate knowledge of vehicle attitude is essential for transforming measurements between reference frames, computing LOS geometry, and generating control commands. Classical attitude-estimation approaches typically rely on gyroscope integration and nonlinear filtering techniques. However, gyroscope drift and sensor uncertainties may degrade estimation quality during long flight intervals.

To overcome these limitations, several studies investigated alternative attitude reconstruction methods based on accelerometers, GNSS measurements, and gravity-vector estimation. In particular, gravity-assisted attitude determination techniques that combine inertial and satellite information to improve orientation estimation were proposed for accelerometer/GNSS-based estimators [19] and further hybridized for ballistic-projectile navigation, guidance, and control [20]. These studies demonstrated that attitude observability can be improved by exploiting additional geometric constraints derived from onboard measurements. Related research on GNSS-based attitude determination has also highlighted the importance of carrier-phase observations, being addressed through single-difference carrier-phase estimation [21], a comprehensive survey of GNSS attitude-determination techniques [22], multi-antenna baseline-ambiguity estimation [23], and, more recently, primary-baseline-switching attitude determination for multi-antenna platforms [24].

Carrier-phase GNSS techniques have become particularly attractive because they enable highly accurate attitude estimation using known antenna baselines. However, their effectiveness depends on reliable integer ambiguity resolution. Ambiguity estimation remains one of the most challenging problems in GNSS navigation, especially under dynamic conditions where satellite visibility, multipath effects, and measurement noise continuously vary; this is addressed through the LAMBDA method and its performance on short baselines [25], its extension to the GNSS compass problem [26], reliable multi-frequency integer resolution using statistical a priori attitude knowledge [27], triple-frequency rapid ambiguity resolution [28], and a broader review of triple-frequency GNSS ambiguity resolution [29]. Recent advances in multi-frequency GNSS receivers and precise-point-positioning techniques have improved ambiguity resolution performance: comparative single-difference and multi-GNSS ambiguity-resolution time-transfer results are reported in [30], multi-frequency BDS-3 augmented real-time positioning in [31], low-cost dual-frequency real-time precise point positioning in [32], and carrier-phase-disciplined real-time GPS oscillators in [33]; yet robustness under highly dynamic rocket conditions remains an open research topic.

In parallel with developments in navigation sensors, artificial intelligence and machine learning techniques have experienced remarkable growth in aerospace applications. Neural networks have been successfully employed for system identification and state estimation, tracing back to the original perceptron formulation [34] and to recurrent architectures with long short-term memory [35], and more recently for trajectory prediction, anomaly detection, and adaptive control through reviewed LSTM-based network architectures [36] and a broader treatment of RNN/LSTM fundamentals [37]. Their ability to approximate highly nonlinear functions makes them attractive for aerospace systems where analytical modeling may be difficult or computationally expensive.

Within the navigation domain, neural networks have been applied to INS/GNSS integration through dynamic-neural-network vehicular navigation [38] and motion-constrained BP-neural-network GNSS/INS integration [39], to multipath mitigation via CNN-LSTM short-baseline positioning [40], to GNSS-outage compensation via CNN-LSTM performance correction [41] and LSTM-based pseudo-measurement aiding [42], and to trajectory prediction using embedded convolutional LSTM networks [43] and deep generative convolutional-recurrent architectures [44]. Similar approaches have also been investigated for spacecraft dynamics, navigation, and control [45], satellite telemetry anomaly detection via feature-attention LSTM networks [46], and autonomous aerial systems through reinforcement-learning-based drone control [47]. These studies consistently demonstrate that learning-based techniques can improve robustness against nonlinear disturbances and partially modeled dynamics.

The present study belongs to a sequence of progressively richer architectures developed for the same application. Model-based GNSS/IMU/SAL hybridization for artillery-rocket guidance was first assessed in [18]. A later neural hybridization method used GNSS/IMU and quadrant-detector signals to estimate terminal LOS information and evaluated its effect on closed-loop impact dispersion [48]. Neural processing was subsequently applied to carrier-phase ambiguity resolution for multi-antenna GNSS attitude determination [49]. Most recently, two neural estimators—one for GNSS double-difference ambiguities and one for body-frame gravity—were embedded in an attitude-restoration chain complemented by SAL observations and compared with a real-time kinematic (RTK) reference [50]. These studies are direct methodological precursors of the present paper.

The preceding configurations did not simultaneously represent ambiguity support, gravity reconstruction, and a dedicated residual LOS-correction estimator as three explicit neural modules within the same GNC information flow. The present work addresses this integration gap and evaluates the three modules over a common set of nominal and combined-disturbance trajectories.

The proposed framework integrates GNSS observations, IMU measurements, and LOS information obtained from a semi-active laser quadrant detector. Three neural modules are incorporated as auxiliary estimators for carrier-phase ambiguity candidates, body-frame gravity reconstruction, and navigation-frame LOS residual correction. Unlike a monolithic data-driven controller, the networks do not command the vehicle directly: their outputs are subject to physical consistency checks and are supplied to a conventional guidance and control chain. The complete architecture is evaluated in a nonlinear six-degree-of-freedom closed loop including aerodynamic coupling, sensor uncertainties, actuator dynamics, and dispersive conditions.

The earlier papers establish methodological continuity and delimit the present contribution. Their impact and attitude results provide historical context, whereas the common closed-loop campaign reported here evaluates the integrated three-module architecture. Circular error probable (CEP) denotes the radius containing 50% of impact points and is used only when discussing impact-dispersion results.

The main contributions of this work can be summarized as follows:

- Closed-loop integration of three explicit neural estimators for GNSS ambiguity support, body-frame gravity reconstruction, and SAL-aided LOS residual correction within a common GNSS/IMU/SAL GNC architecture.
- A physics-consistent fusion and fallback architecture that combines complementary GNSS, inertial, gravity, and SAL information, reducing dependence on any single estimation source while preserving the conventional GNC chain.
- Formulation of the LOS network as a residual correction to the normalized GNSS/IMU LOS, using the transformed SAL observation and vehicle kinematics while retaining the conventional guidance law.
- Evaluation of the integrated architecture over 500 six-degree-of-freedom trajectories organized into five common operating scenarios, including simultaneous sensor, aerodynamic, and environmental disturbances.
- A traceable comparison with the preceding model-based and neural architectures, clarifying the methodological progression to the present three-module configuration.

Table 1 summarizes this progression and identifies the principal level of evidence reported by each study.

The remainder of the paper is organized as follows. Section 2 presents the overall architecture of the proposed neural-network-assisted GNSS/IMU/SAL guidance system, including the onboard sensor configuration, reference frames, and information flow between the different navigation, estimation, guidance, and control subsystems. Section 3 introduces the nonlinear six-degree-of-freedom rocket model together with the kinematic relationships, aerodynamic formulation, actuator representation, and sensor models employed throughout the study. Section 4 describes the proposed neural-network-assisted sensor-fusion methodology, including the ambiguity-support estimator for GNSS carrier-phase processing, the gravity-vector reconstruction network for attitude enhancement, and the LOS-correction module for terminal guidance. Section 5 presents the proportional-navigation guidance law using the corrected LOS estimate and the associated control-allocation strategy used to generate the aerodynamic control commands. Section 6 provides the validation framework and discusses the performance of the individual neural modules as well as the complete integrated navigation and guidance architecture under the modeled six-degree-of-freedom flight conditions. Finally, Section 8 summarizes the main findings of the work and outlines future research directions for neural-network-assisted guidance, navigation, and control systems for precision-guided rockets.

## 2. System Overview

The considered platform is a canard-controlled guided ballistic rocket with fixed aft stabilizing fins, equipped with an inertial measurement unit (IMU), a GNSS receiver or antenna array, and a semi-active laser (SAL) seeker based on a quadrant photodetector. The vehicle follows a ballistic trajectory during boost and mid-course flight and introduces terminal corrections through the actuated forward canards. The proposed configuration is intended for precision-guided rocket applications, where sensor quality, onboard computation, volume, and mass are more limited than in larger tactical missile systems.

The onboard navigation subsystem combines inertial and satellite-based information to estimate position, velocity, attitude, and target-relative geometry throughout the trajectory. The IMU provides high-rate accelerations and angular-rate measurements, which are available independently of external infrastructure and are well suited to capture short-term vehicle dynamics. However, inertial measurements are affected by bias, vibration, scale-factor errors, and accumulated drift. GNSS measurements compensate for these limitations by providing globally referenced position and velocity information with bounded long-term error. When a multi-antenna configuration is available, carrier-phase GNSS observations can also be exploited for attitude determination through baseline geometry and ambiguity resolution.

The terminal sensing subsystem is based on a SAL quadrant detector. During the final portion of the trajectory, an external designator illuminates the target and the onboard seeker detects the reflected laser energy. The displacement of the laser spot on the detector plane provides direct angular information about the target line of sight (LOS) in seeker coordinates. This measurement is particularly valuable close to impact, because LOS estimates derived only from relative GNSS/IMU positioning become increasingly sensitive to small position errors as the target range decreases.

**Figure 1:**
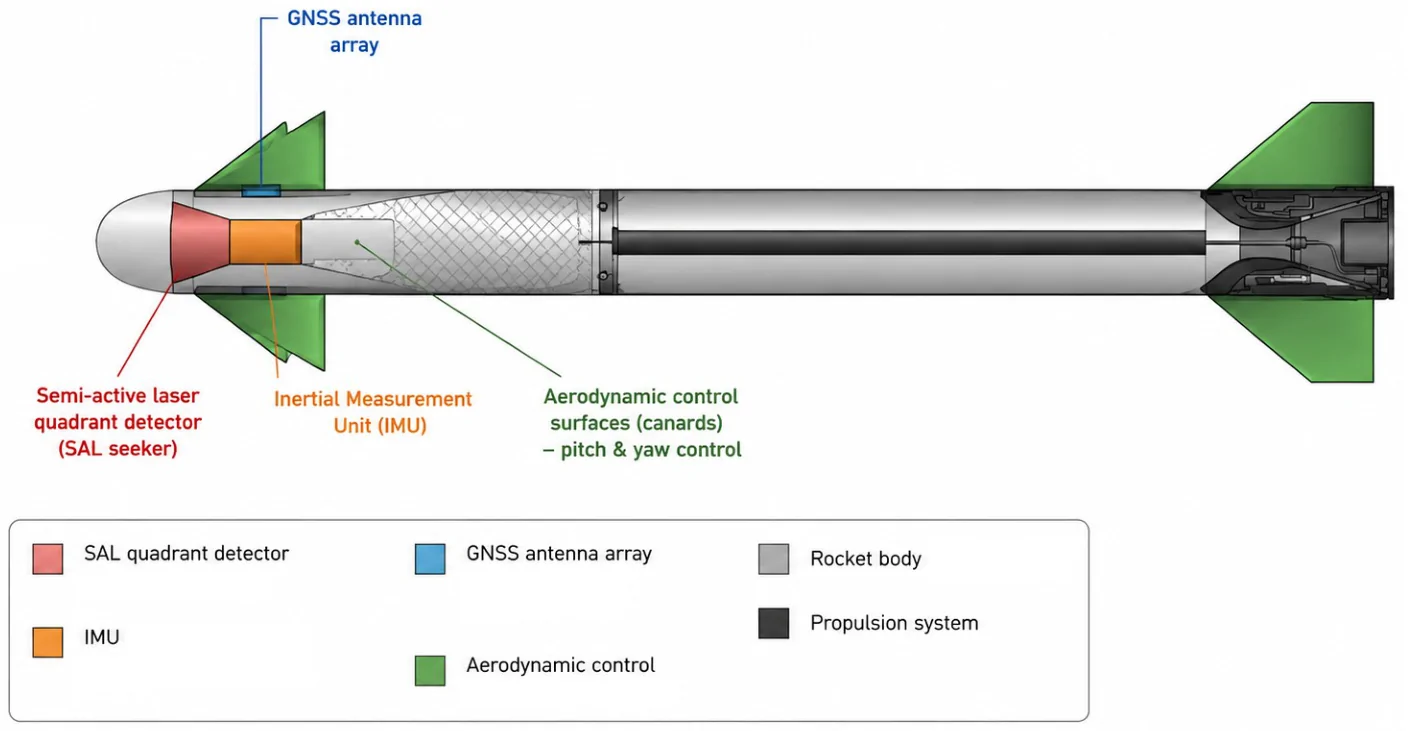
Reference rocket configuration and onboard sensor arrangement.

Figure 1 shows the reference rocket configuration. The IMU and GNSS subsystems are assumed to be active during the complete flight, while the SAL seeker contributes primarily during terminal engagement, when the reflected signal is available and the target is inside the seeker field of view.

**Figure 2:**
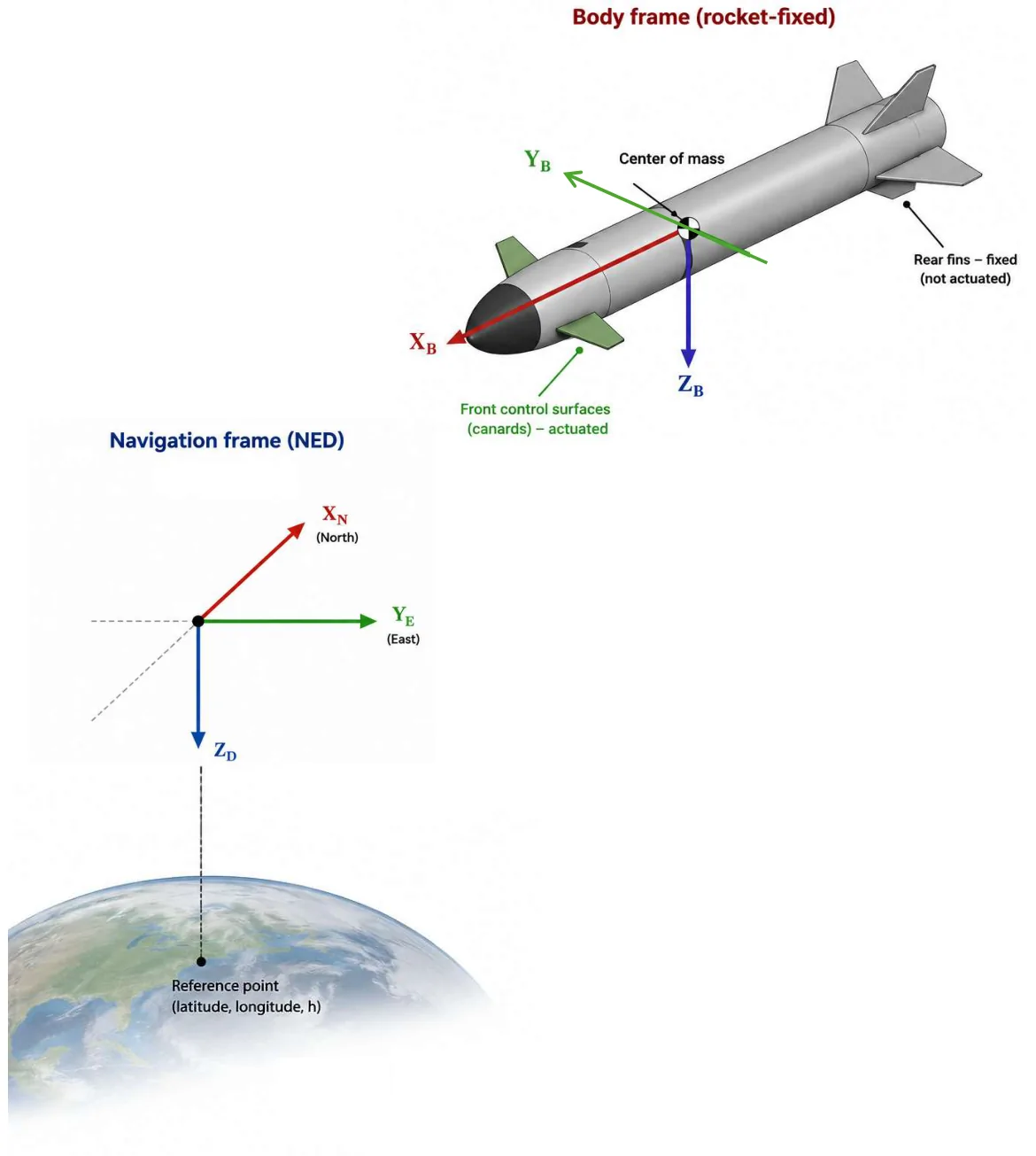
Reference coordinate systems employed by the proposed navigation and guidance architecture.

The proposed framework employs two reference frames: a local navigation frame and a body-fixed frame attached to the rocket. Figure 2 illustrates the coordinate systems used throughout the navigation, guidance, and control architecture.

The navigation frame is defined as a conventional North-East-Down (NED) reference frame, denoted by $F_{n}=X_{N},Y_{E},Z_{D}$. This frame is used to represent the vehicle position, velocity, target location, and line-of-sight (LOS) geometry. GNSS measurements are naturally expressed in this frame after the corresponding coordinate transformations.

The body frame, denoted by $F_{b}=X_{B},Y_{B},Z_{B}$, is rigidly attached to the rocket and centered at its center of mass. The $X_{B}$ axis is aligned with the longitudinal axis of the vehicle and points toward the nose of the rocket. The $Y_{B}$ axis points toward the starboard side of the vehicle, while the $Z_{B}$ axis completes a right-handed coordinate system. Consequently, attitude is defined by the rotation between the NED frame and the body frame.

IMU measurements, including specific forces and angular rates, are obtained in body coordinates, whereas GNSS measurements are referenced to the navigation frame. Therefore, accurate attitude estimation is required to consistently transform information between both reference systems and to reconstruct the LOS vector employed by the terminal guidance law. Throughout this work, all guidance, navigation, and control computations are formulated using these two reference frames.

**Figure 3:**
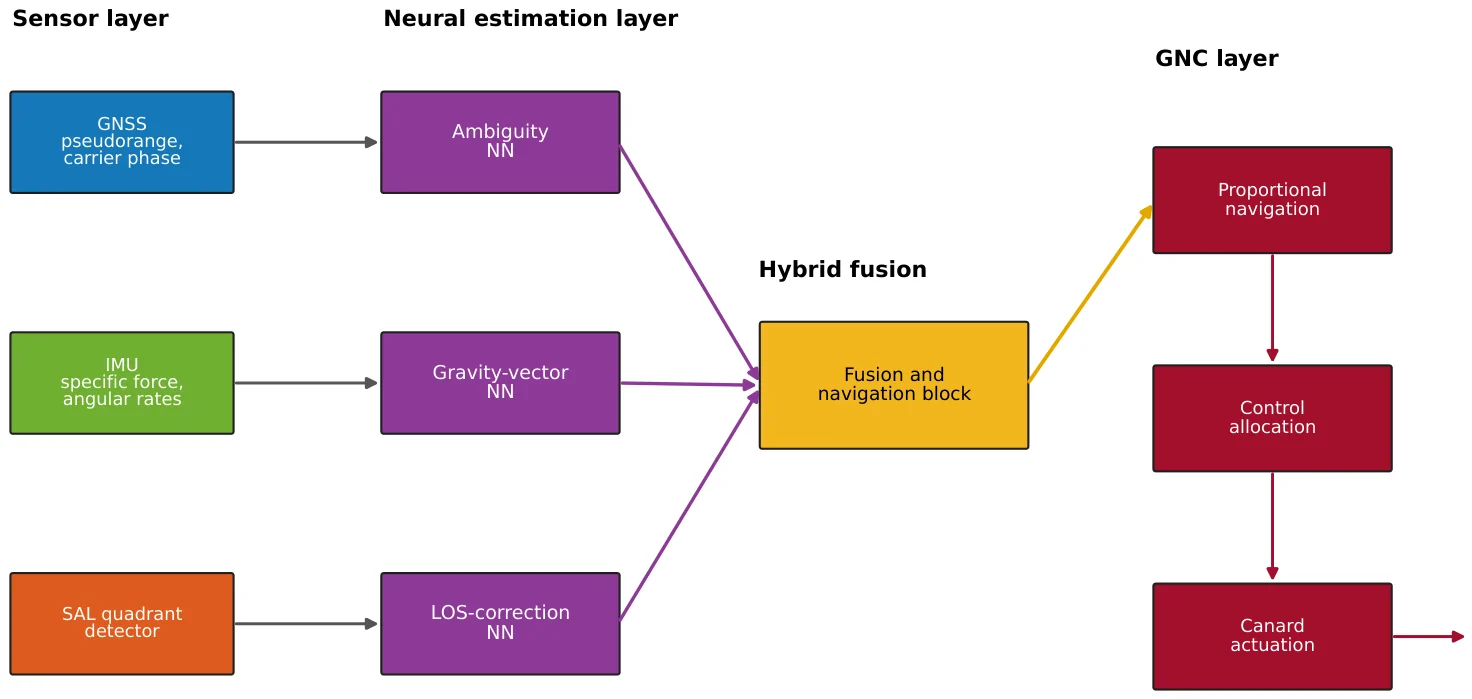
Neural-network-assisted GNSS/IMU/SAL fusion architecture for rocket guidance, navigation, and control.

Figure 3 presents the overall information flow of the proposed architecture. The system combines conventional model-based estimation with neural-network-assisted modules designed to support nonlinear estimation tasks that are difficult to solve robustly using purely analytical models. The neural estimators correct intermediate navigation quantities, which are physically validated before entering the conventional guidance and control chain; they never command the rocket directly.

The architecture is organized into four hierarchical layers. The first layer is the sensor layer, which includes GNSS pseudorange, Doppler, and carrier-phase measurements; IMU accelerations and angular-rate measurements; and quadrant-detector spot-displacement signals. Unlike conventional schemes that rely only on processed navigation outputs, the proposed framework can exploit raw and derived measurements to increase the information available for state estimation.

The second layer is the neural estimation layer. Three specialized neural modules are considered. The first supports GNSS carrier-phase ambiguity estimation by learning nonlinear relationships among satellite geometry, phase observables, and admissible ambiguity states. The second reconstructs gravity-vector information from inertial measurements and auxiliary flight variables, providing an additional attitude reference when gyroscopic integration is degraded. The third improves LOS consistency by correcting discrepancies between GNSS/IMU-derived target geometry and SAL quadrant-detector measurements during terminal flight.

The third layer is the fusion and navigation layer. In this block, neural outputs are combined with physical measurement equations and dynamic constraints to obtain estimates of position, velocity, attitude, LOS geometry, and target-relative states. The fusion process is therefore hybrid: it preserves the interpretability of model-based navigation while exploiting the approximation capability of neural networks for sensor nonlinearities, ambiguity-support information, and terminal LOS corrections.

The fourth layer is the guidance, navigation, and control layer. The fused state and corrected LOS estimates are passed to a conventional proportional-navigation law, followed by control allocation adapted to the rocket dynamics. The resulting guidance commands are converted into canard deflections while accounting for actuator dynamics, aerodynamic coupling, and flight-envelope limitations.

**Figure 4:**
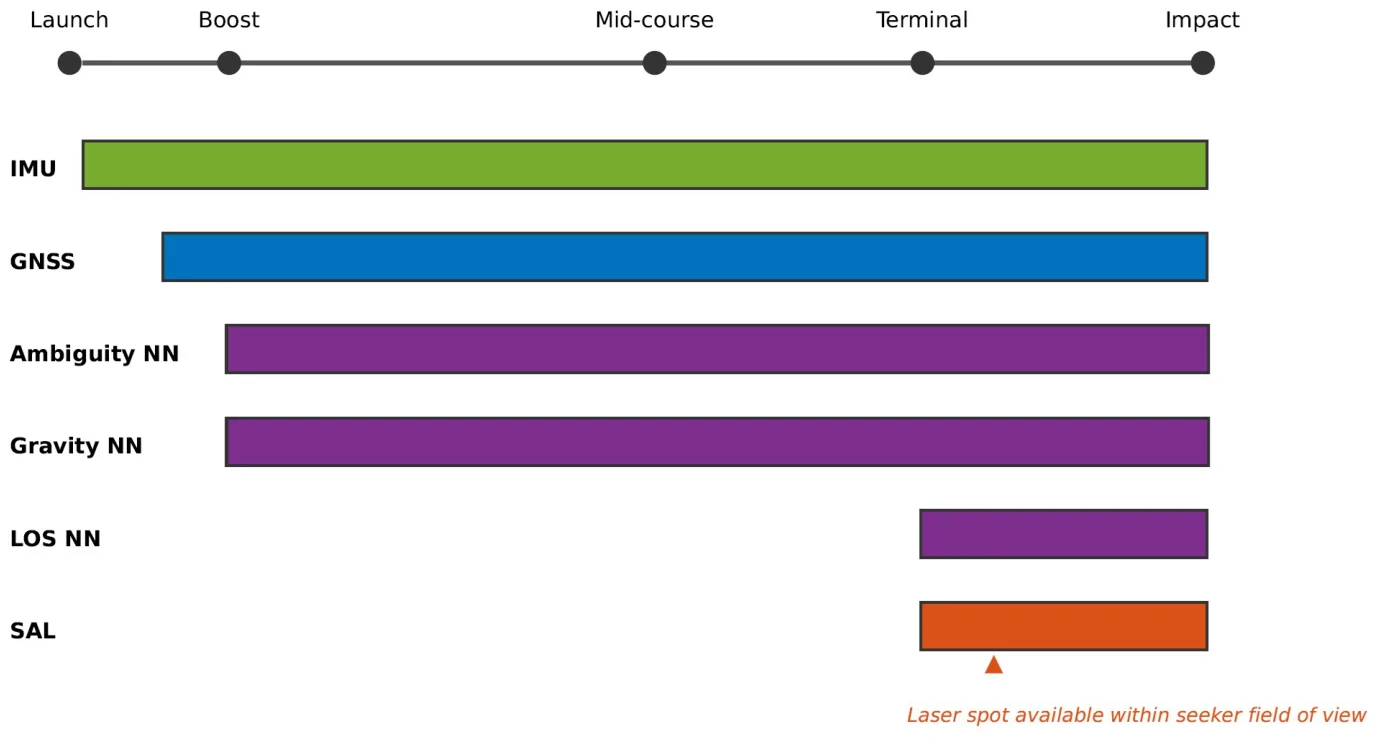
Conceptual availability of GNSS, IMU, neural-estimation, and SAL measurements along the rocket trajectory.

The temporal availability of each sensing modality is shown in Figure 4. GNSS and IMU measurements support the navigation solution from launch to impact. Neural estimators can operate when their required input signals are available, although their relative importance changes along the trajectory. The ambiguity and gravity networks require only GNSS and IMU signals and are therefore available from launch to impact, whereas the LOS-correction network additionally requires the SAL observation and is consequently available only during the Terminal phase. The SAL detector is mainly exploited during terminal flight, where it supplies direct angular target information.

**Figure 5:**
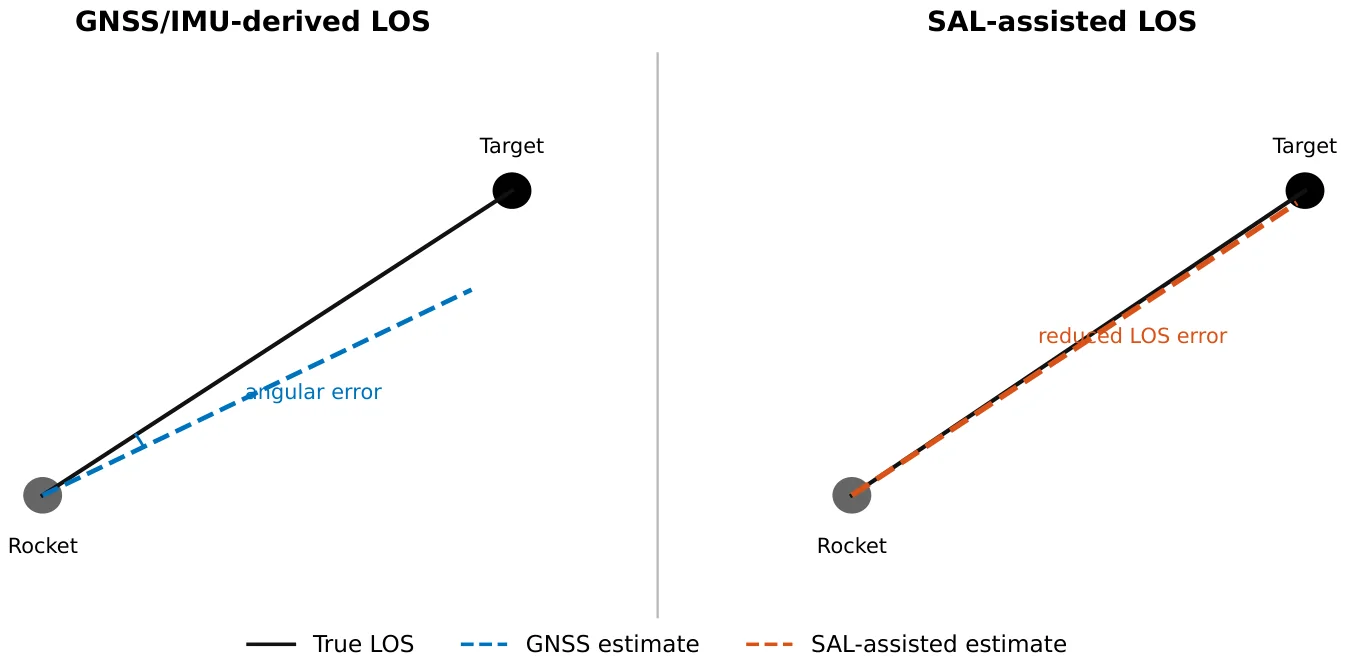
Conceptual comparison between GNSS/IMU-derived LOS estimation and SAL-assisted LOS estimation during terminal engagement.

Figure 5 illustrates the terminal motivation for introducing SAL-assisted fusion. When the LOS is reconstructed only from GNSS/IMU relative position estimates, small position errors may generate significant angular deviations close to impact. The SAL quadrant detector provides a direct angular measurement of the illuminated target and therefore reduces the sensitivity of the guidance law to accumulated navigation drift.

From a system perspective, the proposed architecture integrates three complementary research lines. The first is GNSS-based attitude estimation and ambiguity support using carrier-phase measurements. The second is onboard attitude and LOS reconstruction through neural sensor fusion. The third is hybrid GNSS/IMU and SAL guidance for terminal rocket navigation. The present study operates these elements concurrently in the propagated closed loop and measures how their errors change under a common set of disturbances.

## 3. Dynamic, Kinematic, and Sensor Models

This section presents the mathematical models employed to describe the rocket motion, the onboard sensing architecture, and the geometric relationships required for multisensor fusion. Particular emphasis is placed on the quantities required by the proposed neural-network-assisted estimation framework, namely, vehicle attitude, GNSS carrier-phase observables, gravity-vector information, and terminal line-of-sight measurements obtained from the semi-active laser seeker.

### 3.1. Reference Frames and Kinematics

As introduced in Section 2, the navigation and body frames are formalized as follows:

The attitude of the vehicle is represented through the direction cosine matrix

(1) $$C_{b}^{n},$$

which transforms vectors from body coordinates into navigation coordinates. Consequently,

(2) $$v^{n}=C_{b}^{n}v^{b}.$$

The relative position between the target and the rocket is given by

(3) $$r_{rel}^{n}=r_{T}^{n}-r_{R}^{n},$$

where $r_{T}^{n}$ and $r_{R}^{n}$ denote the target and rocket positions expressed in the NED frame.

The corresponding line-of-sight vector is

(4) $$L^{n}=\frac{r_{rel}^{n}}{∥r_{rel}^{n}∥}.$$

The LOS vector expressed in body coordinates becomes

(5) $$L^{b}={\left(C_{b}^{n}\right)}^{T}L^{n}.$$

The LOS rate required by the guidance law is obtained either from relative kinematics or directly from the SAL measurements during terminal engagement.

### 3.2. Six-Degree-of-Freedom Rocket Dynamics

The rocket is modeled as a rigid body with six degrees of freedom. The translational motion in the navigation frame is governed by

(6) $$m{\dot{v}}^{n}=F_{T}^{n}+F_{A}^{n}+F_{C}^{n}+mg^{n},$$

where *m* denotes the vehicle mass, $F_{T}^{n}$ is the thrust force, $F_{A}^{n}$ represents the aerodynamic forces generated by the rocket body and fixed stabilizing fins, $F_{C}^{n}$ corresponds to the control forces produced by the actuated canards, and $g^{n}$ is the gravity vector.

The equations shown here use a local, non-rotating, flat-Earth NED approximation over the simulated engagement; Earth-rotation and transport-rate terms are therefore neglected in this reduced presentation. Mass and inertia are time-dependent during motor burn and are denoted by m(t) and J(t) in the simulation, although the explicit time argument is suppressed below for readability.

The kinematic equations are

(7) $${\dot{r}}^{n}=v^{n}.$$

The rotational dynamics are described by the angular-momentum balance

(8) $$J{\dot{\omega }}^{b}+\dot{J}\omega^{b}+\omega^{b}\times J\omega^{b}=M_{A}^{b}+M_{C}^{b},$$

where J is the inertia tensor, $\omega^{b}$ is the body angular-rate vector, $M_{A}^{b}$ denotes aerodynamic moments, and $M_{C}^{b}$ contains the moments generated by the actuated canards. The $\dot{J}\omega^{b}$ term accounts for the prescribed inertia variation during motor burn and vanishes when inertia is constant.

Attitude is propagated with the unit quaternion $q={\left[q_{0},q_{v}^{T}\right]}^{T}$ according to

(9) $$\dot{q}=\frac{1}{2}\left[\begin{array}{cc} 0 & -{\left(\omega^{b}\right)}^{T}\\ \omega^{b} & -[\omega^{b}\times ] \end{array}\right]q,\;∥q∥=1,$$

where $\left[\omega^{b}\times \right]$ is the skew-symmetric matrix satisfying $\left[\omega^{b}\times \right]u=\omega^{b}\times u$ for any vector u. Numerical renormalization is applied after each integration step.

The aerodynamic force and moment models are expressed as

(10) $$F_{A}^{b}=\frac{1}{8}\rho {∥v^{b}∥}^{2}\pi d^{2}\left[\begin{array}{c} C_{X}\\ C_{Y}\\ C_{Z} \end{array}\right],$$

(11) $$M_{A}^{b}=\frac{1}{8}\rho {∥v^{b}∥}^{2}\pi d^{3}\left[\begin{array}{c} C_{l}\\ C_{m}\\ C_{n} \end{array}\right],$$

where ρ is the local air density, $v^{b}$ is the rocket velocity expressed in body coordinates, and *d* is the rocket reference diameter.

The aerodynamic force coefficients $\left(C_{X},C_{Y},C_{Z}\right)$ and moment coefficients $\left(C_{l},C_{m},C_{n}\right)$ depend on the flight condition, including Mach number, angle of attack, sideslip angle, body angular rates, and control-surface deflections.

The vehicle employs two independent control channels generated by the actuated forward canards: a vertical deflection channel, denoted by $\delta_{v}$, and a horizontal deflection channel, denoted by $\delta_{h}$. These control inputs generate additional aerodynamic forces and moments, while the rear fins remain fixed and provide passive aerodynamic stability.

The control-force vector is modeled as

(12) $$F_{C}^{b}=\frac{1}{8}\rho {∥v^{b}∥}^{2}\pi d^{2}\left(\left[\begin{array}{c} C_{X_{\delta_{h}}}\\ C_{Y_{\delta_{h}}}\\ C_{Z_{\delta_{h}}} \end{array}\right]\delta_{h}+\left[\begin{array}{c} C_{X_{\delta_{v}}}\\ C_{Y_{\delta_{v}}}\\ C_{Z_{\delta_{v}}} \end{array}\right]\delta_{v}\right),$$

where $\delta_{h}$ and $\delta_{v}$ represent the horizontal and vertical control deflections, respectively, and the corresponding aerodynamic derivatives quantify the force increments generated by each control channel.

Similarly, the moments generated by the canards are expressed as

(13) $$M_{C}^{b}=\frac{1}{8}\rho {∥v^{b}∥}^{2}\pi d^{3}\left(\left[\begin{array}{c} C_{l_{\delta_{h}}}\\ C_{m_{\delta_{h}}}\\ C_{n_{\delta_{h}}} \end{array}\right]\delta_{h}+\left[\begin{array}{c} C_{l_{\delta_{v}}}\\ C_{m_{\delta_{v}}}\\ C_{n_{\delta_{v}}} \end{array}\right]\delta_{v}\right),$$

where $\left(C_{l_{\delta_{h}}},C_{m_{\delta_{h}}},C_{n_{\delta_{h}}}\right)$ and $\left(C_{l_{\delta_{v}}},C_{m_{\delta_{v}}},C_{n_{\delta_{v}}}\right)$ are the control-moment derivatives associated with the horizontal and vertical canard commands.

Under the assumption of small control deflections, the total control contribution is obtained as the linear superposition of the effects generated by both control channels. This formulation allows the guidance law to command independent pitch and yaw corrections while maintaining compatibility with the nonlinear six-degree-of-freedom flight-dynamics model.

These forces and moments are incorporated into the six-degree-of-freedom equations of motion and determine the translational and rotational response of the rocket throughout the flight envelope.

### 3.3. IMU Measurement Model

The IMU provides specific-force and angular-rate measurements at high update rates.

Accelerometer outputs are modeled as

(14) $${\tilde{f}}^{b}=f^{b}+b_{a}+n_{a},$$

where $b_{a}$ represents accelerometer biases and $n_{a}$ denotes measurement noise. Under the local non-rotating navigation-frame approximation, the ideal specific force is

(15) $$f^{b}={\left(C_{b}^{n}\right)}^{T}\left({\dot{v}}^{n}-g^{n}\right).$$

Similarly, gyroscope measurements are represented by

(16) $${\tilde{\omega }}^{b}=\omega^{b}+b_{g}+n_{g}.$$

The measured angular-rate model uses gyroscope bias $b_{g}$ and measurement noise $n_{g}$. Together, the accelerometer and gyroscope measurements constitute the primary input for inertial state propagation and for the gravity-vector neural estimator in Section 4.

### 3.4. GNSS Measurement Model

The GNSS subsystem provides position, velocity, and carrier-phase observations.

The pseudorange measurement associated with satellite *i* is expressed as

(17) $$\rho_{i}=R_{i}+c\left(\delta t_{r}-\delta t_{i}\right)+I_{i}+T_{i}+\epsilon_{\rho ,i},$$

where $R_{i}$ is the geometric range, *c* is the speed of light, $\delta t_{r}$ and $\delta t_{i}$ are receiver and satellite clock offsets, $I_{i}$ and $T_{i}$ denote ionospheric and tropospheric delays, and $\epsilon_{\rho ,i}$ collects pseudorange noise and residual errors.

Carrier-phase observations are modeled as

(18) $$\Phi_{i}=R_{i}+c\left(\delta t_{r}-\delta t_{i}\right)+T_{i}-I_{i}+\lambda N_{i}+\epsilon_{\Phi ,i},$$

where $\Phi_{i}$ is expressed in meters, $N_{i}$ is the integer ambiguity in cycles, and λ is the carrier wavelength. Residual multipath, phase-centre, and unmodeled effects are included in $\epsilon_{\Phi ,i}$. The ionospheric contribution has the opposite sign to the code observation when both equations are expressed in range units.

For attitude processing, receiver and satellite clock terms are removed by the single- and double-difference formulation used for neural ambiguity support in [49] and subsequently incorporated into the onboard sensor-fusion architecture of [50]. The neural module operates on those differenced carrier-phase quantities and their associated geometry rather than interpreting an undifferenced raw phase as an integer baseline observation.

These ambiguities constitute one of the variables estimated through the neural-support architecture proposed in this work. The carrier-phase observations are not only employed for positioning purposes but also constitute the basis for the attitude-determination process adopted in this work. The accelerometer/GNSS vector-correspondence formulation is described in [19], while neural candidate generation for carrier-phase ambiguity resolution is developed in [49]. In the present architecture, the accepted ambiguities are used to reconstruct the baselines between the GNSS antennas. The resulting baseline vectors provide geometric constraints for estimating the attitude matrix $C_{b}^{n}$ relating the body and navigation frames. Consequently, attitude accuracy depends directly on ambiguity quality, which motivates the neural ambiguity-support module described in Section 4. The GNSS-derived attitude estimate is subsequently combined with inertial information and gravity-vector reconstruction to improve robustness under highly dynamic flight conditions.

### 3.5. Semi-Active Laser Quadrant Detector Model

During terminal flight, the target is illuminated by an external laser designator and the reflected energy is captured by the onboard quadrant detector.

Let *A*, *B*, *C*, and *D* denote the four detector outputs. The normalized spot coordinates are computed as

(19) $$u=\frac{\left(A+D)-(B+C\right)}{A+B+C+D},$$

(20) $$v=\frac{\left(A+B)-(C+D\right)}{A+B+C+D}.$$

The corresponding LOS angles are obtained through seeker calibration functions

(21) $$\theta_{LOS}=f_{u}\left(u\right),\;\psi_{LOS}=f_{v}\left(v\right).$$

Here, $f_{u}$ and $f_{v}$ are the pre-calibrated mappings from normalized detector coordinates to the two seeker angular coordinates $\theta_{LOS}$ and $\psi_{LOS}$.

These measurements provide direct angular information regarding target location and therefore complement the LOS estimate reconstructed from GNSS and inertial measurements.

### 3.6. State Vector for Sensor Fusion

The complete state vector employed by the proposed architecture is defined as

(22) $$x={\left[\begin{array}{ccccccc} r^{n} & v^{n} & q & \omega^{b} & b_{a} & b_{g} & L^{n} \end{array}\right]}^{T},$$

where q denotes the attitude quaternion representation and $L^{n}$ is the target LOS expressed in the navigation frame. Biases and attitude are propagated by the model-based navigation layer; the displayed state vector defines the information exchanged with the neural estimators rather than a single end-to-end neural state.

The neural estimation modules introduced in Section 4 provide auxiliary information regarding gravity-vector reconstruction, GNSS ambiguity support, and LOS correction, which is subsequently incorporated into the fusion process to improve navigation consistency during terminal guidance.

## 4. Neural Network-Assisted Sensor Fusion

The proposed fusion framework combines model-based navigation with neural-network-assisted estimation modules. The neural networks are not used to command the vehicle directly. Instead, they operate as auxiliary estimators that improve intermediate navigation quantities before these quantities are passed to the guidance and control subsystem. This approach preserves the physical interpretation of the GNC architecture while exploiting the ability of neural networks to approximate nonlinear sensor relationships.

The architecture includes three specialized neural modules. The first module supports GNSS carrier-phase ambiguity estimation. The second module reconstructs gravity-related information in body axes to improve attitude observability. The third module corrects the line-of-sight estimate during terminal engagement by combining GNSS/IMU and semi-active laser measurements. The fusion logic includes geometric and dynamic consistency checks before a neural output is accepted.

### 4.1. Common Dataset Generation Framework and Simulation Environment

The three neural estimators are trained on data generated by the same nonlinear six-degree-of-freedom simulation environment and share a common data-generation methodology, which is described once here and referenced from the network-specific subsections that follow.

*Simulation environment.* The nonlinear six-degree-of-freedom flight-dynamics model, the sensor models of Section 3, and the guidance, control-allocation, and actuator models of Section 5 are implemented in a MATLAB/Simulink environment. The rigid-body equations of motion are integrated with a fixed-step fourth-order Runge–Kutta (RK4) scheme at a 1 ms integration step. The three neural networks are implemented and trained in Python using TensorFlow/Keras, on a workstation equipped with a multi-core CPU and a single consumer-grade GPU; training and inference use double-precision floating-point arithmetic.

The closed-loop and dataset-generation simulations were run in MATLAB/Simulink R2025b on Windows 11 Education using an Intel Core i9-13900K CPU. The three networks were implemented and trained in Python 3.10 with TensorFlow 2.13/Keras on the same workstation, equipped with a single NVIDIA RTX 4090 GPU. Each of the 4000 development trajectories and 500 evaluation trajectories was assigned a deterministic trajectory-generation seed, recorded with its trajectory index and used for the trajectory and sensor-noise random-number generators. Fixed training random-number-generator states were used for weight initialization and mini-batch shuffling. The recorded trajectory seeds and training random-number-generator states permit deterministic regeneration of the datasets and trained models.

*Common trajectory generation.* Training and evaluation trajectories are generated by independently sampling launch conditions, aerodynamic-coefficient uncertainty, wind, vehicle attitude, angular rates, sensor-noise realizations, and GNSS-degradation periods, as detailed in Section 3. Each trajectory is assigned a unique random seed, and all samples belonging to one trajectory are kept together when forming the training, validation, and test subsets (Table 2), preventing time-correlated leakage between subsets. The development dataset comprises 4000 trajectories, independent of the 500 trajectories used in the closed-loop evaluation campaign of Section 6. Network-specific sampling rates and feature vectors are described in the corresponding dataset-generation paragraphs below.

Because the three estimators share the feedforward architecture of Table 3 (three hidden layers of 64 units each with ReLU activations), the number of trainable parameters for input dimension $d_{in}$ and output dimension $d_{out}$ is $\left(d_{in}+1\right)\times 64+2\times \left(64+1\right)\times 64+\left(64+1\right)\times d_{out}$. The gravity-vector network (9 inputs and 3 outputs) therefore contains 9155 trainable parameters, and the LOS-correction network (12 inputs and 3 outputs) contains 9347. For the fixed-size ambiguity-support configuration described here, m=16 double-difference channels give 64 inputs and 16 outputs, corresponding to 13,520 trainable parameters. This fixed 64-input/16-output layout is the one actually implemented and trained, matching the adopted three-antenna, eight-to-ten-satellite channel configuration; satellite channels that are temporarily unavailable or degraded do not alter this dimensionality, since the fixed channel ordering used to build $z_{GNSS}$ is preserved and channel availability is instead flagged through the corresponding entries of the signal-quality vector. The complete three-network architecture consequently contains 32,022 trainable parameters. These counts establish the storage and arithmetic scale of the estimators but do not, by themselves, demonstrate real-time feasibility on flight hardware.

The sensor-noise, GNSS-degradation, SAL, guidance, and actuation settings used consistently in dataset generation and closed-loop evaluation are summarized in Table 4.

### 4.2. Neural Support for GNSS Ambiguity Resolution

Carrier-phase observations provide high relative precision under favorable tracking conditions and therefore constitute a key element for attitude determination using multi-antenna configurations. The undifferenced observation follows Equation (18); for attitude processing, receiver–satellite and antenna differencing removes the common clock terms and leads to a double-difference carrier-phase model of the form

(23) $$\Phi_{DD,j}=R_{DD,j}+T_{DD,j}-I_{DD,j}+\lambda N_{DD,j}+\varepsilon_{\Phi ,DD,j},$$

where the subscript DD denotes the selected reference-antenna/reference-satellite double-difference convention, $R_{DD,j}$ is the differenced geometric range, $T_{DD,j}$ and $I_{DD,j}$ are the corresponding atmospheric-delay terms, λ is the carrier wavelength, $N_{DD,j}$ is the integer ambiguity, and $\varepsilon_{\Phi ,DD,j}$ collects residual carrier-phase errors.

The correct estimation of the ambiguity vector

(24) $$N_{DD}={\left[\begin{array}{cccc} N_{DD,1} & N_{DD,2} & \cdots & N_{DD,m} \end{array}\right]}^{T}$$

is essential for high-accuracy baseline reconstruction and attitude determination.

Although ambiguity resolution is traditionally performed through algorithms such as the least-squares ambiguity decorrelation adjustment (LAMBDA), reliable fixing becomes more difficult when the underlying measurements are degraded by limited visibility, poor geometry, cycle slips, or multipath. These effects are particularly relevant in guided rocket applications, where rapid attitude variations and high angular rates introduce significant measurement variability.

To provide a data-driven initialization, a neural network is incorporated as an ambiguity-support estimator. Rather than replacing integer validation, the network acts as a preprocessing stage that produces ambiguity candidates that must subsequently be checked using conventional residual, geometry, and integer-consistency tests. The regression head is trained by minimizing mean squared error (MSE); its output is an ambiguity candidate, not a calibrated confidence value.

#### 4.2.1. Ambiguity Dataset Generation

The training dataset follows the common generation framework of Section 4.1. In addition to the shared trajectory variations, the ambiguity dataset specifically covers satellite constellation geometry, carrier-phase and pseudorange measurement noise, baseline orientation, multipath disturbances, and GNSS signal interruptions (Table 4). For each simulated scenario, the true ambiguity vector is known and therefore provides the desired network target.

The complete dataset is divided into training (70%), validation (15%), and testing subsets (15%). Because samples within one simulated trajectory are strongly time-correlated, complete trajectories and their random seeds are assigned exclusively to one subset. The 70/15/15 partition is therefore performed at trajectory level, preventing near-adjacent states from the same realization from appearing in both development and test subsets. The development dataset is generated from 4000 independently seeded six-degree-of-freedom trajectories, distinct from the 500 trajectories reserved for the closed-loop evaluation campaign of Section 6. The ambiguity network is sampled at the 10 Hz GNSS update rate over the flight, the gravity network is sampled at the 200 Hz IMU update rate per trajectory, and the LOS network is sampled at the 100 Hz SAL update rate over the terminal SAL-active window only, which is why the three networks have different total sample counts despite sharing the same trajectory pool and the same 70/15/15 trajectory-level split. Flight duration is not fixed across the dataset: because each trajectory is initialized with a different randomized seed, its total flight time differs slightly from trajectory to trajectory, with all trajectories spanning approximately 15 to 20 s of flight from launch to impact.

**Figure 6:**
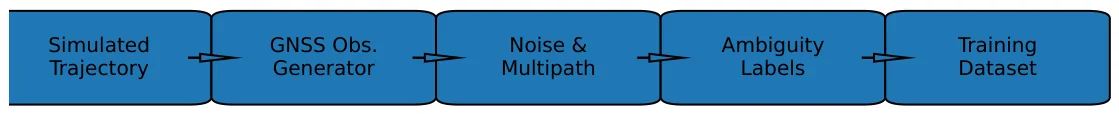
Dataset generation process for GNSS ambiguity estimation.

Figure 6 summarizes the generation process used to obtain the ambiguity-estimation dataset.

#### 4.2.2. Ambiguity Feature Extraction

The input vector is composed of carrier-phase and pseudorange information together with descriptors characterizing the quality of the GNSS solution. The resulting feature vector is

(25) $$z_{GNSS}={\left[\begin{array}{cccc} \rho_{DD}^{T}\; & \Phi_{DD}^{T}\; & d_{sat}^{T}\; & s_{q}^{T} \end{array}\right]}^{T},$$

where

- $\rho_{DD}$ contains differenced pseudorange-derived quantities;
- $\Phi_{DD}$ contains differenced carrier-phase observables;
- $d_{sat}$ contains satellite-geometry indicators;
- $s_{q}$ contains signal-quality metrics.

All networks use fixed-dimensional inputs. GNSS observables follow the deterministic satellite and measurement ordering used during dataset generation, while signal interruptions and changing availability are represented consistently through the signal-quality descriptors in $s_{q}$. The same convention is applied to the training, validation, and test trajectories.

Prior to training, all features are normalized to zero mean and unit variance.

#### 4.2.3. Ambiguity-Support Network Architecture

The ambiguity-support estimator employs a fully connected feedforward neural network. The architecture consists of an input layer, several hidden layers using nonlinear activation functions, and an output layer providing ambiguity estimates.

The network mapping is represented by

(26) $${\hat{N}}_{DD}=f_{N}\left(z_{GNSS}\right),$$

where ${\hat{N}}_{DD}$ contains the estimated double-difference ambiguity candidates.

**Figure 7:**
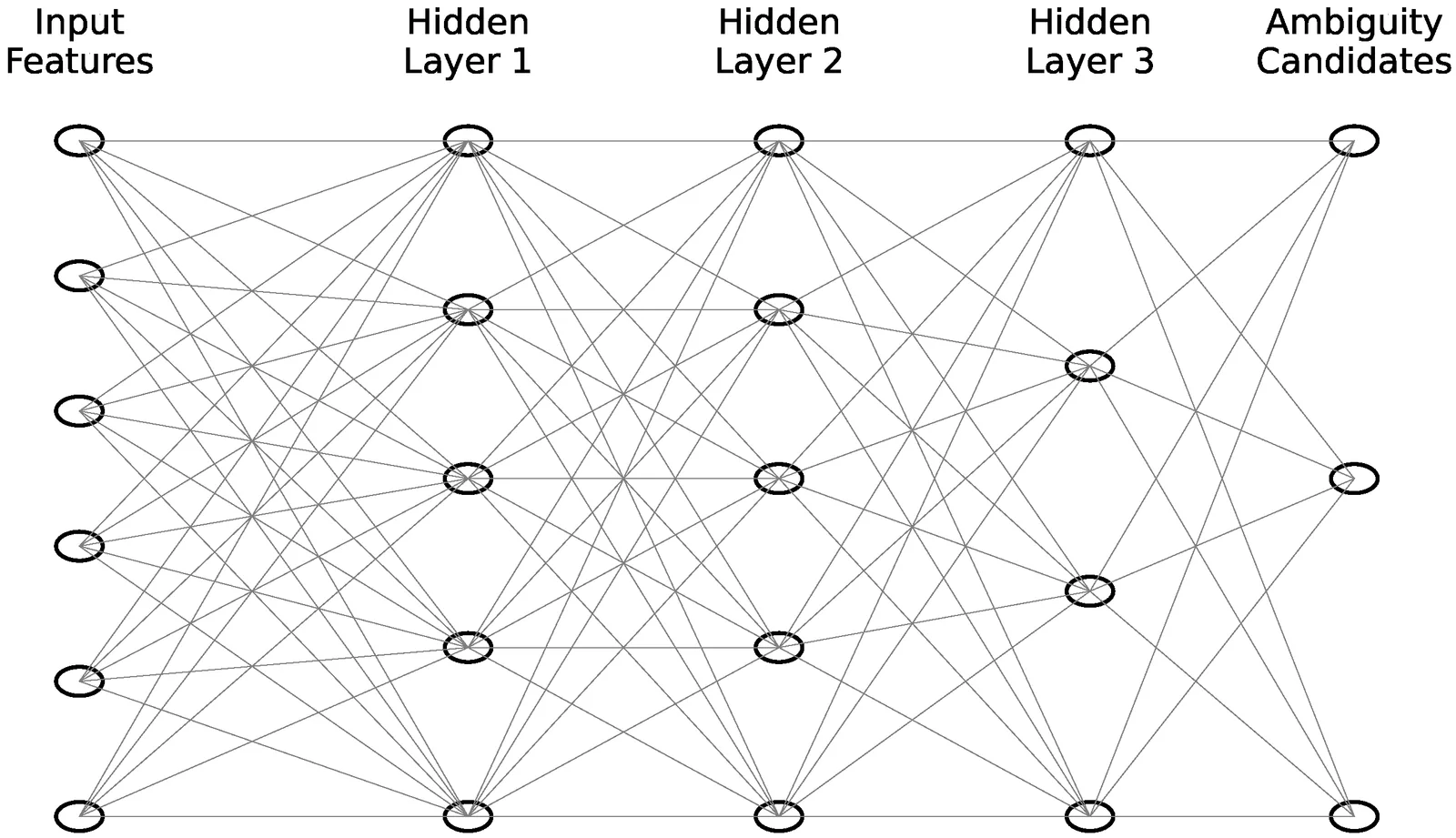
Neural-network architecture employed for ambiguity support estimation.

Figure 7 illustrates the neural architecture.

The principal training hyperparameters are summarized in Table 3.

#### 4.2.4. Ambiguity-Support Network Training

The network parameters are obtained through supervised learning. For each training sample, the neural network predicts an ambiguity vector ${\hat{N}}_{DD}$, which is compared with the corresponding true ambiguity vector $N_{DD}$. The resulting prediction error is used to update the network weights through the optimization procedure described below.

The loss function employed during training is

(27) $$J=\frac{1}{M}\sum_{k=1}^{M}{∥N_{DD,k}-{\hat{N}}_{DD,k}∥}^{2},$$

where *M* denotes the number of training samples.

Training is performed until convergence of the validation error. Early-stopping criteria are employed to avoid overfitting and improve generalization capability.

#### 4.2.5. Integration with the Navigation Architecture

The ambiguity-support estimator is not intended to replace conventional ambiguity-resolution methods. Instead, the network provides ambiguity candidates that are subsequently incorporated into the carrier-phase processing chain and accepted only after physical and measurement-consistency checks.

The resulting accepted ambiguity estimates are used by the attitude-determination subsystem defined in Section 3 and therefore contribute to estimation of the transformation matrix $C_{b}^{n}$. In this role, the network supplies a candidate-generation mechanism while preserving the physical GNSS measurement model and conventional validation stage.

### 4.3. Gravity-Vector Reconstruction and Attitude Support

The second neural module estimates the gravity vector expressed in body coordinates. An ideal accelerometer measures specific force rather than gravity directly; reconstructing this vector therefore requires separating gravitational orientation from translational acceleration and sensor errors using the available kinematic information. During highly dynamic flight, this inverse relationship is difficult to approximate with the sensors and imperfect models.

The proposed neural estimator reconstructs the gravity vector in body coordinates directly from onboard measurements. This information is subsequently incorporated into the attitude-determination process as an additional geometric constraint.

#### 4.3.1. Gravity-Reconstruction Dataset Generation

**Figure 8:**
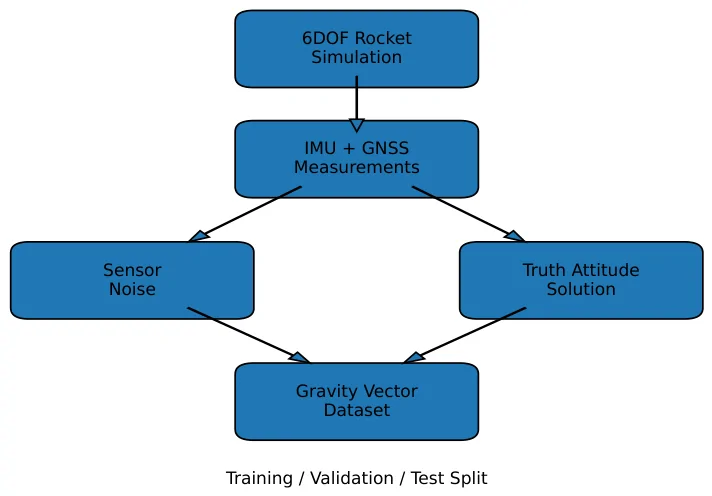
Dataset generation process for gravity-vector reconstruction.

The training dataset follows the common generation framework of Section 4.1, using the nonlinear six-degree-of-freedom simulation model defined in Section 3. For each simulated trajectory, the true vehicle attitude is available and therefore the exact gravity vector expressed in body coordinates can be computed. The resulting dataset contains synchronized inertial, GNSS, and truth-model information. Figure 8 summarizes the generation process employed to construct the gravity-reconstruction dataset.

The complete dataset is divided into training, validation, and testing subsets following the same trajectory-level partition strategy employed for the ambiguity-support network (Table 2).

#### 4.3.2. Gravity-Network Input Features

The input vector employed by the network is defined as

(28) $$z_{g}={\left[\begin{array}{ccc} {\tilde{f}}^{bT}\; & {\tilde{\omega }}^{bT}\; & v^{bT} \end{array}\right]}^{T},$$

where ${\tilde{f}}^{b}$ denotes the measured specific force, ${\tilde{\omega }}^{b}$ is the measured angular-rate vector, and $v^{b}$ is the rocket velocity supplied by the navigation subsystem and expressed in body coordinates. Because transforming velocity into body axes depends on the current attitude solution, $v^{b}$ is a coupled navigation input rather than an independent direct measurement. During closed-loop operation, it is formed exclusively with the online attitude estimate; truth attitude is used only to generate supervised labels and evaluation errors.

Prior to training, all features are normalized using the mean and standard deviation computed from the training set.

#### 4.3.3. Gravity-Reconstruction Network Architecture

A fully connected feedforward neural network is employed for gravity-vector reconstruction. The network receives the feature vector $z_{g}$ and produces an estimate of the gravity vector in body coordinates.

The mapping performed by the network is represented as

(29) $${\hat{g}}^{b}=f_{g}\left(z_{g}\right),$$

where ${\hat{g}}^{b}$ is the estimated gravity vector.

**Figure 9:**
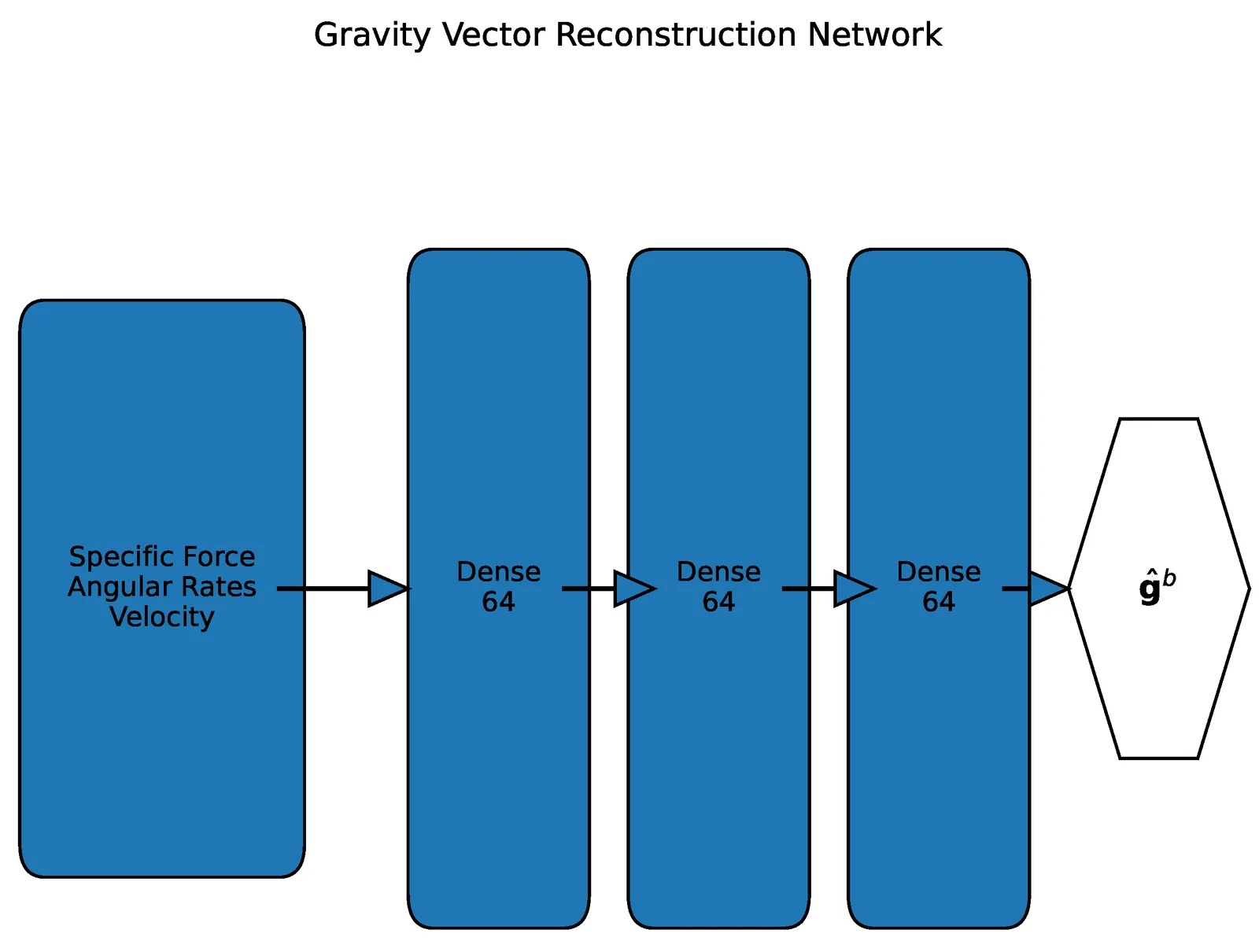
Neural-network architecture employed for gravity-vector reconstruction.

The architecture employed in this work is illustrated in Figure 9.

The network architecture and training hyperparameters are identical to those employed for the ambiguity-support estimator (Table 3), ensuring a consistent neural-network design throughout the proposed fusion framework.

#### 4.3.4. Gravity-Reconstruction Network Training

The network parameters are obtained through supervised learning. For each training sample, the network prediction ${\hat{g}}^{b}$ is compared with the corresponding true gravity vector $g^{b}$ extracted from the simulation truth model.

The loss function employed during training is

(30) $$J=\frac{1}{M}\sum_{k=1}^{M}{∥g_{k}^{b}-{\hat{g}}_{k}^{b}∥}^{2},$$

where *M* denotes the number of training samples.

Early stopping and validation monitoring are employed to reduce overfitting and improve generalization capability.

#### 4.3.5. Attitude Reconstruction

Once the gravity vector has been estimated, it can be paired with the known gravity direction in the navigation frame,

(31) $$g^{n}={\left[\begin{array}{ccc} 0 & 0 & g \end{array}\right]}^{T},$$

where g>0 is the local gravitational-acceleration magnitude in the Down direction of the NED frame. This provides an additional vector correspondence between body and navigation coordinates.

The accelerometer/GNSS vector-correspondence principle of [19] and its ballistic-projectile hybridization in [20] are used to combine the estimated gravity vector with GNSS-derived attitude information and inertial measurements to reconstruct the attitude matrix $C_{b}^{n}$. The resulting vector correspondence supplements gyroscope propagation and GNSS baseline information; the present campaign reports gravity-vector error rather than a separate incremental attitude-error comparison.

### 4.4. Laser-Aided Line-of-Sight Correction

During terminal flight, the line-of-sight estimate becomes one of the most critical quantities for guidance. Although GNSS and inertial measurements provide accurate navigation information during most of the trajectory, the LOS estimate derived from the navigation solution becomes increasingly sensitive to small position errors as the target range decreases.

A preliminary LOS vector can be reconstructed from the GNSS/IMU navigation solution as

(32) $$L_{GI}^{n}=\frac{r_{T}^{n}-r_{R}^{n}}{∥r_{T}^{n}-r_{R}^{n}∥}.$$

where $r_{T}^{n}$ and $r_{R}^{n}$ denote the target and rocket positions expressed in the navigation frame, and the subscript GI identifies the GNSS/IMU geometric estimate.

The semi-active laser quadrant detector provides an independent angular measurement of the target direction during terminal engagement. Using the calibration functions defined in Section 3, the detector outputs are converted into a LOS estimate expressed in body coordinates $L_{SAL}^{b}.$

For fusion with the navigation-frame geometric LOS, the seeker observation is transformed using the online attitude estimate,

(33) $$L_{SAL}^{n}=C_{b}^{n}L_{SAL}^{b}.$$

This transformation makes the LOS module dependent on the current attitude solution and therefore couples it to the ambiguity and gravity modules in closed loop. The matrix $C_{b}^{n}$ is obtained from the online attitude estimate; truth attitude is used only to construct supervised targets and evaluation errors, not as a network input during closed-loop operation.

The availability of two independent LOS estimates provides the basis for the neural correction strategy proposed in this work.

The distinction from the earlier neural LOS hybridization in [48] is the residual formulation used here. The previous approach predicted detector-plane/LOS quantities directly from GNSS/IMU and SAL signals; the present network estimates a navigation-frame correction to an already normalized GNSS/IMU geometric LOS and then enforces unit norm before guidance use.

#### 4.4.1. LOS-Correction Dataset Generation

The training dataset follows the common generation framework of Section 4.1. In addition to the shared trajectory variations, the LOS dataset specifically covers engagement geometry, target range, detector noise, optical misalignments, and intermittent SAL visibility conditions (Table 4). For each simulated trajectory, the exact LOS vector is available from the truth model and therefore constitutes the desired network target.

The dataset partition follows the same training, validation, and testing strategy adopted for the ambiguity-support network (Table 2).

#### 4.4.2. LOS-Network Input Features

The neural LOS-correction module receives information from both sensing modalities together with additional flight-condition variables.

The input feature vector is defined as

(34) $$z_{L}={\left[\begin{array}{cccc} {\left(L_{GI}^{n}\right)}^{T}\; & {\left(L_{SAL}^{n}\right)}^{T}\; & {\left(v^{n}\right)}^{T}\; & {\left(\omega^{b}\right)}^{T} \end{array}\right]}^{T}.$$

These features contain geometric, kinematic, and attitude-related information that allows the network to learn systematic discrepancies between both LOS sources.

#### 4.4.3. LOS-Correction Network Architecture

A fully connected feedforward neural network is employed to estimate LOS residual corrections. The network architecture follows the same design philosophy adopted for the ambiguity-support and gravity-reconstruction estimators. Consequently, the same hyperparameter configuration summarized in Table 3 is employed.

The network mapping is represented as

(35) $$\Delta L^{n}=f_{L}\left(z_{L}\right),$$

where $\Delta L^{n}$ denotes the estimated LOS residual.

#### 4.4.4. LOS-Correction Network Training

The network parameters are obtained through supervised learning. For each training sample, the network first predicts the residual $\Delta L^{n}=f_{L}\left(z_{L}\right)$ of Equation (35). This residual is then combined with $L_{GI}^{n}$ and renormalized exactly as in the piecewise definition of Equation (37) below to obtain the reconstructed estimate ${\hat{L}}^{n}$, and it is this reconstructed, unit-norm quantity (not the raw residual) that is compared against the truth-model LOS vector during training, so that the network is optimized end to end through the normalization step. The loss function employed during training is therefore

(36) $$J=\frac{1}{M}\sum_{k=1}^{M}{∥L_{true,k}^{n}-{\hat{L}}_{k}^{n}∥}^{2},$$

where *M* denotes the number of training samples and ${\hat{L}}_{k}^{n}$ is the reconstructed estimate of Equation (37), obtained from the residual $\Delta L_{k}^{n}$ of that same sample.

As in the previous neural modules, early stopping and validation monitoring are employed to reduce overfitting and improve generalization performance.

#### 4.4.5. LOS Reconstruction

The corrected LOS estimate is obtained through the following case-based definition, which makes the previously implicit rejection logic explicit:

(37) $${\hat{L}}^{n}=\left\{\begin{array}{cc} \frac{L_{GI}^{n}+\Delta L^{n}}{∥L_{GI}^{n}+\Delta L^{n}∥}, & ∥L_{GI}^{n}+\Delta L^{n}∥>\epsilon_{L},\\ L_{GI}^{n}, & ∥L_{GI}^{n}+\Delta L^{n}∥\leq \epsilon_{L}, \end{array}\right.$$

where $\epsilon_{L}>0$ is a small numerical tolerance that prevents division by a near-zero norm (set to $\epsilon_{L}=10^{-3}$ throughout the evaluation campaign, Section 4). For $∥L_{GI}^{n}+\Delta L^{n}∥>\epsilon_{L}$, normalization enforces unit norm; otherwise, the correction is rejected and the uncorrected geometric LOS is retained.

The resulting LOS estimate combines the global geometric consistency provided by GNSS/IMU navigation with the direct angular information supplied by the semi-active laser seeker while preserving compatibility with the subsequent guidance law.

### 4.5. Hybrid Fusion Logic

The outputs of the three neural modules are integrated into the navigation layer together with the conventional state estimates. The hybrid fusion vector can be written as

(38) $$x_{F}={\left[\begin{array}{cccc} x^{T} & {\hat{N}}_{DD}^{T} & {\left({\hat{g}}^{b}\right)}^{T} & {\left({\hat{L}}^{n}\right)}^{T} \end{array}\right]}^{T},$$

where x denotes the conventional navigation state vector.

#### Model-Based Navigation Filter

The “model-based” half of the hybrid architecture referred to throughout this section is a closed-form error-state extended Kalman filter (EKF) that propagates the navigation state between accepted neural or measurement updates and that the fallback logic reverts to whenever a neural output is rejected. The filter state is the 15-element error vector

(39) $$\delta x={\left[\begin{array}{ccccc} \delta \theta^{T} & \delta v^{nT} & \delta r^{nT} & \delta b_{a}^{T} & \delta b_{g}^{T} \end{array}\right]}^{T},$$

where δθ is the attitude error (small-angle) vector, and $\delta v^{n}$, $\delta r^{n}$, $\delta b_{a}$, $\delta b_{g}$ are the velocity, position, accelerometer-bias, and gyroscope-bias errors, respectively. Between measurement updates, the nominal state is propagated by strapdown mechanization of the IMU outputs of Section 3 (Equations (14)–(16)), and the error covariance is propagated as

(40) $$P_{k}^{-}=\Phi_{k-1}P_{k-1}^{+}\Phi_{k-1}^{T}+Q_{k-1},$$

where $\Phi_{k-1}$ is the discrete-time state-transition matrix obtained from the linearized error-state dynamics and $Q_{k-1}$ is the process-noise covariance derived from the accelerometer and gyroscope noise densities of Table 4.

Accepted GNSS position and velocity solutions enter as a direct linear measurement,

(41) $$z_{GNSS,k}=\left[\begin{array}{c} r_{GNSS,k}^{n}-{\hat{r}}_{k}^{n-}\\ v_{GNSS,k}^{n}-{\hat{v}}_{k}^{n-} \end{array}\right]=H_{GNSS}\;\delta x_{k}+n_{GNSS,k},$$

where $H_{GNSS}$ is the constant 6×15 selection matrix that extracts the position- and velocity-error blocks from $\delta x_{k}={\left[\delta \theta^{T}\;\delta v^{nT}\;\delta r^{nT}\;\delta b_{a}^{T}\;\delta b_{g}^{T}\right]}^{T}$ (an identity block in the velocity and position columns, zero elsewhere). The accepted double-difference ambiguity candidates ${\hat{N}}_{DD}$ (Section 4) are converted, after integer validation, into a baseline-derived attitude pseudo-measurement that enters through the same update equation with an attitude-only observation matrix. The accepted gravity-vector estimate ${\hat{g}}^{b}$ enters as an attitude-constraint measurement through the linearized cross-product sensitivity

(42) $$z_{g,k}={\hat{g}}_{k}^{b}-{\left({\hat{C}}_{b,k}^{n-}\right)}^{T}g^{n}\approx \left[{\left({\hat{C}}_{b,k}^{n-}\right)}^{T}g^{n}\times \right]\delta \theta_{k}+n_{g,k},$$

where $n_{g,k}$ has covariance set from the empirical gravity-reconstruction error of Section 6.4. Every measurement update follows the standard EKF gain, state, and covariance correction,

(43) $$K_{k}=P_{k}^{-}H_{k}^{T}{\left(H_{k}P_{k}^{-}H_{k}^{T}+R_{k}\right)}^{-1},\;\delta {\hat{x}}_{k}^{+}=K_{k}\;z_{k},\;P_{k}^{+}=\left(I-K_{k}H_{k}\right)P_{k}^{-},$$

after which the error state is injected into the nominal attitude, velocity, position, and bias estimates and reset to zero. This filter, not the neural outputs, is the estimate retained by the fallback logic of the next subsection whenever a neural candidate fails a consistency check.

Before being accepted by the guidance subsystem, the neural outputs are checked against physical constraints. These checks include admissible ambiguity ranges, unit-norm LOS constraints, attitude-orthogonality constraints, measurement-availability conditions, and available sensor-quality indicators. An output that violates a hard constraint is rejected and the model-based navigation value of Section Model-Based Navigation Filter is retained. The numerical thresholds used throughout the evaluation campaign are as follows: an admissible double-difference ambiguity range of ±8 cycles about the network candidate (values outside this range are rejected outright); the normalization tolerance $\epsilon_{L}=10^{-3}$ in Equation (37); an attitude-orthogonality tolerance of ${∥C_{b}^{n\;T}C_{b}^{n}-I∥}_{F}\leq 10^{-2}$; and a minimum GNSS carrier-to-noise-density ratio of 28 dB-Hz for a candidate to be considered for acceptance. These thresholds were fixed a priori from the sensor-noise budget of Table 4 and are not adjusted per scenario. Acceptance, rejection, and fallback-substitution rates per module and per scenario are reported in Section 6.6.

After the ±8-cycle admissible search, the best integer candidate is accepted only when the LAMBDA ratio of the second-best to the best conditional sum of squared residuals is at least 3.0. A false fix is an accepted integer ambiguity vector for which at least one component differs from the corresponding true integer ambiguity vector. Gravity-network outputs are accepted only when the attitude estimate obtained with the candidate gravity vector satisfies ${∥C_{b}^{n\;T}C_{b}^{n}-I∥}_{F}\leq 10^{-2}$ and $∥{\hat{g}}^{b}∥$ lies within ±5% of the reference local gravity magnitude *g* defined in the vehicle model. LOS epochs without an available SAL measurement are excluded from the LOS-module acceptance-rate denominator, which is restricted to the SAL-active terminal window. Outside that window, the guidance law uses the uncorrected geometric LOS by design rather than as a fallback event.

The fusion sequence is summarized below:

1. Acquire GNSS and IMU observations and SAL measurements when available.
2. Build the GNSS feature vector and generate ambiguity candidates with the neural ambiguity module.
3. Propagate the model-based navigation solution and estimate the body-frame gravity vector.
4. Update the attitude solution using the accepted GNSS and gravity-vector constraints.
5. Compute the GNSS/IMU-derived LOS. When SAL is available, transform its LOS observation to navigation axes, estimate the neural residual, and normalize the corrected LOS.
6. Apply geometric and measurement-consistency checks; rejected neural outputs fall back to the corresponding model-based value.
7. Supply the resulting attitude, LOS, and navigation quantities to guidance and control.

The resulting architecture retains model-based propagation and guidance while introducing neural approximations at three intermediate estimation stages. The quantitative evidence associated with each stage is stated separately in Section 6.

## 5. Guidance and Control Formulation

The fused navigation solution generated by the proposed GNSS/IMU/SAL architecture is employed to construct the terminal guidance commands. In particular, the corrected LOS estimate obtained from the neural fusion layer constitutes the primary input to the guidance law. Since the objective of the terminal phase is to minimize miss distance without exceeding the available control authority, the guidance formulation must account for the highly nonlinear rocket dynamics and the rapid variation of engagement geometry close to impact.

### 5.1. Terminal Engagement Geometry

Let

(44) $$r_{rel}^{n}=r_{T}^{n}-r_{R}^{n}$$

denote the relative position vector between the target and the rocket.

The corresponding geometric LOS unit vector is

(45) $$L^{n}=\frac{r_{rel}^{n}}{∥r_{rel}^{n}∥}.$$

The relative velocity vector is defined as

(46) $$v_{rel}^{n}=v_{T}^{n}-v_{R}^{n}.$$

The closing velocity is obtained from

(47) $$V_{c}=-{\left(v_{rel}^{n}\right)}^{T}L^{n}.$$

A positive value of $V_{c}$ indicates that the rocket is approaching the target.

### 5.2. Proportional Navigation with Neural-Corrected LOS

Classical proportional navigation generates acceleration commands proportional to LOS angular velocity and closing velocity. For conventional missiles, this strategy provides excellent interception performance under a broad range of engagement conditions.

Guided rockets additionally exhibit significant body rotation, strong aerodynamic coupling, limited control authority, actuator saturation, and rapidly varying aerodynamic coefficients during terminal flight. In the present architecture, these effects are addressed by the downstream control allocation and nonlinear plant model. The equation below retains the conventional proportional-navigation structure; the modification considered in this paper is the use of the neural-corrected LOS and its physical projection before the command is allocated to the canards.

The commanded lateral acceleration is therefore expressed as

(48) $$a_{c}^{n}=NV_{c}{\dot{\hat{L}}}^{n},$$

where *N* denotes the navigation constant and ${\dot{\hat{L}}}^{n}$ is the navigation-frame LOS rate computed from the corrected estimate ${\hat{L}}^{n}$ generated by the neural fusion layer.

To avoid direct finite differencing of a noisy unit vector, the LOS rate is computed from the relative-velocity projection when the corresponding navigation quantities are available,

(49) $${\dot{\hat{L}}}^{n}=\frac{1}{\hat{R}}\left(I-{\hat{L}}^{n}{\left({\hat{L}}^{n}\right)}^{T}\right){\hat{v}}_{rel}^{n},\;\hat{R}=∥{\hat{r}}_{rel}^{n}∥.$$

Here, I is the 3×3 identity matrix, hats identify online navigation estimates, and $\hat{R}$ is the estimated target range. This expression enforces orthogonality between the unit LOS and its derivative. The navigation constant, terminal-guidance activation condition, LOS-rate filtering, maximum canard deflection, and canard-rate limit are held fixed across all five evaluation scenarios so that the disturbance class is the only scenario-level change in the guidance and actuation configuration.

The use of the corrected LOS estimate is one of the principal differences with respect to a GNSS/IMU-only guidance architecture. Incorporating the SAL observation provides a direct angular source during terminal engagement and reduces reliance on relative position alone.

### 5.3. Acceleration Command Generation

The commanded acceleration vector is first transformed into body coordinates through

(50) $$a_{c}^{b}={\left(C_{b}^{n}\right)}^{T}a_{c}^{n}.$$

Only the lateral acceleration components are employed for terminal steering, since the longitudinal component is primarily governed by propulsion and aerodynamic drag.

The desired lateral acceleration vector is therefore defined as

(51) $$a_{lat}^{b}={\left[\begin{array}{ccc} 0 & a_{Y,c} & a_{Z,c} \end{array}\right]}^{T}.$$

These commands represent the accelerations required to reduce LOS error and drive the rocket toward the designated target.

### 5.4. Control Allocation

The generated lateral-acceleration commands are mapped directly to canard deflections through a locally linearized control-effectiveness model. No separate attitude-command loop is introduced in the reduced allocation equation below.

Assuming a locally linearized control effectiveness model, the relationship between commanded accelerations and control deflections may be approximated as

(52) $$\delta_{c}=B^{†}\left(a_{lat}^{b}-a_{0}^{b}\right),$$

where

(53) $$\delta_{c}={\left[\begin{array}{cc} \delta_{h} & \delta_{v} \end{array}\right]}^{T}$$

contains the horizontal and vertical canard commands, $B^{†}$ is the Moore–Penrose pseudoinverse of the control-effectiveness matrix B, and $a_{0}^{b}$ represents the predicted body-frame lateral acceleration at zero canard deflection.

The control-effectiveness matrix is obtained from the aerodynamic derivatives introduced in Section 3 and therefore varies with flight condition, dynamic pressure, Mach number, and angle of attack.

### 5.5. Actuator Constraints

The canard actuators are subject to both position and rate limitations. Consequently, the commanded deflections satisfy

(54) $$|\delta_{h}|\leq \delta_{max},\;\left|\delta_{v}\right|\leq \delta_{max},$$

and

(55) $$|{\dot{\delta }}_{h}|\leq {\dot{\delta }}_{max},\;\left|{\dot{\delta }}_{v}\right|\leq {\dot{\delta }}_{max}.$$

Here, $\delta_{max}$ and ${\dot{\delta }}_{max}$ denote the common deflection and deflection-rate limits, respectively. These constraints are explicitly included in the nonlinear simulation environment to represent finite actuator authority and bandwidth.

### 5.6. Integration with the Neural Fusion Architecture

The complete guidance loop begins with the acquisition of GNSS, IMU, and SAL measurements. The neural fusion layer subsequently generates corrected attitude and LOS estimates. These quantities are employed to compute the LOS rate and closing velocity required by the proportional-navigation law. The resulting acceleration commands are transformed into canard deflections and applied to the six-degree-of-freedom rocket model.

The neural modules therefore modify the navigation quantities used by the guidance law without directly commanding the vehicle or replacing its physical structure. All evaluated closed-loop trajectories completed without numerical divergence; formal stability margins are outside the present scope.

## 6. Simulation Results

The proposed neural-network-assisted GNSS/IMU/SAL architecture combines three estimation modules derived from the preceding research line: a neural ambiguity-support estimator, a gravity-vector reconstruction network, and a residual LOS-correction network. This section evaluates their simultaneous operation as active components of the nonlinear six-degree-of-freedom closed loop.

All results in this section originate from closed-loop runs. At every simulation update, the sensor models generate GNSS, IMU, and, when available, SAL observations; the neural and model-based navigation stages process those observations; the guidance law computes the acceleration command; the control allocation generates canard commands; and the actuator and vehicle models propagate the next state. The reported errors are therefore recorded while estimator outputs affect the subsequent trajectory, rather than being computed by replaying a fixed trajectory in open loop.

The simulation environment includes nonlinear aerodynamics, propulsion, atmospheric disturbances, actuator dynamics, GNSS observations, inertial measurements, and quadrant-detector observations. Five scenario-specific trajectory sets, together comprising the full 500-trajectory evaluation campaign, are used to examine progressively more demanding conditions. In every run, the three neural outputs enter the navigation chain and affect the subsequently propagated vehicle state.

### 6.1. Simulation Environment, Sensor Parameters, and Evaluation Scenarios

The simulation and network-development environment and the sensor, guidance, and actuation parameters are described in Section 4.1 and Table 4. The adopted values correspond to a navigation-grade IMU, a geodetic-grade GNSS receiver, and a precision SAL seeker, and are simulated consistently across every result reported in this section, including the model-based baseline of Section 6.2 and the ablation study of Section 6.8.

Table 5 defines the five evaluation scenarios introduced qualitatively above, making explicit the disturbance parameters that distinguish them.

### 6.2. Baseline Configuration

To assess whether the neural estimators improve on their classical counterparts, a matched conventional baseline was executed as 500 paired trials using identical initial conditions, disturbance realizations, sensor-noise sequences, and random seeds in each configuration. The five disturbance scenarios are those of Table 5; each neural module is replaced by its model-based equivalent while the remainder of the GNC chain (six-degree-of-freedom plant, guidance law, control allocation, and actuator model) is left unchanged:

- *Ambiguity resolution*: the neural candidate generator is replaced by the conventional LAMBDA method operating directly on the double-difference carrier-phase observations of Section 4, followed by the same integer-validation stage used for the neural candidates.
- *Attitude support*: the gravity-vector network is disabled; attitude is propagated by gyroscopic integration corrected only by the GNSS-derived baseline attitude of Section 3, without the additional gravity-vector geometric constraint.
- *LOS estimation*: the LOS-correction network is disabled; the guidance law uses the uncorrected GNSS/IMU geometric LOS $L_{GI}^{n}$ of Section 4 directly, without SAL-based residual correction.

The baseline therefore isolates the contribution of each neural module by reverting it to the corresponding conventional estimator described in Section 3, while retaining the same sensor models, noise levels, disturbance scenarios, and physical fallback logic of Section 4. Baseline results are reported alongside the corresponding neural-configuration results throughout Section 6.3, Section 6.4, Section 6.5, Section 6.6, Section 6.7 and Section 6.8, and the full baseline-versus-three-module comparison is summarized in Section 6.8.

### 6.3. GNSS Ambiguity Estimation Results

The first neural module generates candidates for carrier-phase ambiguities. Accurate ambiguity determination is essential when a multi-antenna GNSS configuration is used for attitude estimation, because an incorrectly accepted integer candidate can produce a large baseline error. As defined in Section 4, the network is a regression-based candidate generator followed by conventional integer validation.

**Figure 10:**
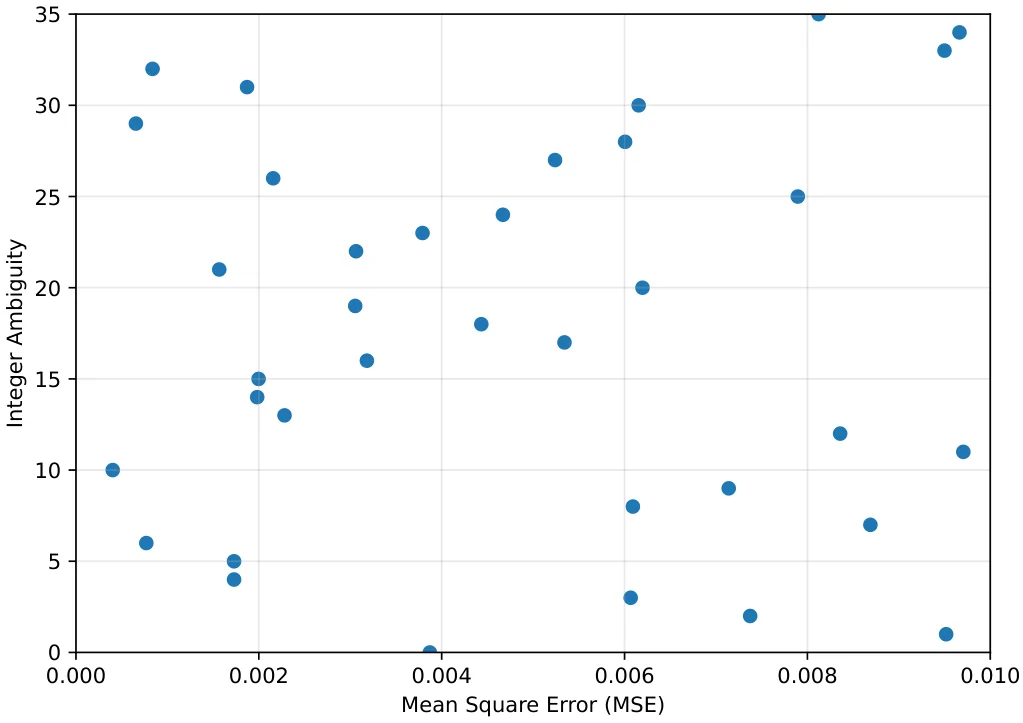
Squared prediction-error distribution of the neural ambiguity-candidate estimator as a function of the corresponding integer ambiguity value.

Figure 10 shows the squared prediction error associated with different ambiguity values and therefore characterizes the regression stage of the candidate generator. Fix success and false-fix probability are properties of the complete candidate-and-validation chain and are not represented by this plot.

Across the displayed integer-ambiguity range, all squared prediction errors lie within the plotted 0–$10^{-2}$ interval and show no evident increase with ambiguity magnitude. Thus, the network supplies bounded regression candidates over the sampled cases; conventional validation remains responsible for accepting an integer fix. A dedicated ambiguity comparison is reported in [49], while the subsequent attitude and multisensor-fusion assessment is reported separately in [50].

Figure 10 characterizes only the regression stage. The complete candidate-and-validation chain is characterized here by its fix success rate (correctly fixed baseline), false-fix rate (an incorrect integer accepted as valid), and rejection rate (no candidate accepted, triggering the model-based fallback of Section Model-Based Navigation Filter), evaluated over the same 500 closed-loop trajectories and compared against the LAMBDA-only baseline of Section 6.2. Table 6 reports these statistics per scenario.

The scenario-specific percentages in Table 6 are computed over the ambiguity-resolution attempts belonging to each scenario. The final row is reported conservatively as the unweighted arithmetic mean of the five scenario-level percentages and is not described as a pooled rate.

The neural candidate generator improves the fix success rate relative to the LAMBDA-only baseline in every scenario, with the largest margin under GNSS degradation and combined disturbances (+2.1 and +3.2 percentage points, respectively), consistent with the intended role of the network as a data-driven prior that aids conventional validation when geometry and signal quality are poor. Under the navigation-grade GNSS receiver of Table 4, the false-fix rate remains below 1.3% in all scenarios for both configurations; rejected candidates fall back to the model-based filter of Section Model-Based Navigation Filter rather than propagating an unvalidated fix.

### 6.4. Gravity Vector Reconstruction and Attitude Estimation

The second neural module reconstructs the gravity vector expressed in body coordinates. This information provides an additional attitude reference that complements both inertial propagation and GNSS-derived attitude estimates.

To evaluate the estimator within the closed loop, the network was exercised using the six-degree-of-freedom trajectories introduced at the beginning of this section. The evaluation campaign includes launch perturbations, aerodynamic uncertainties, wind disturbances, high-angular-rate maneuvers, sensor noise, and GNSS degradation scenarios.

The trajectories were divided into representative flight-condition categories to characterize degradation under progressively more demanding operating conditions.

The *Nominal Flight Conditions* scenario corresponds to trajectories generated using the reference aerodynamic model, nominal atmospheric conditions, nominal sensor characteristics, and the absence of external disturbances. This scenario serves as a baseline for evaluating the intrinsic reconstruction capability of the neural network.

The *Wind Disturbances* scenario introduces steady and gusting wind components acting on the vehicle throughout the trajectory. Wind velocities were randomly generated within the range ±15m/s for each navigation-frame axis, producing variations in angle of attack, sideslip angle, and aerodynamic loading.

The *High Angular-Rate Maneuvers* scenario includes aggressive attitude excursions representative of terminal-guidance corrections. Initial attitude perturbations of up to $\pm 15^{\circ }$ and angular rates of up to $200^{\circ }/s$ were considered in order to increase the inertial contribution measured by the accelerometers.

The *GNSS Degradation Periods* scenario incorporates intermittent reductions in GNSS measurement quality. These conditions were simulated through increased pseudorange noise, degraded satellite geometry, temporary loss of visible satellites, and short GNSS outages lasting up to several seconds.

Finally, the *Combined Disturbances* scenario simultaneously includes wind perturbations, high angular-rate maneuvers, sensor-noise increases, GNSS degradation periods, and aerodynamic-coefficient uncertainties. This is the most demanding modeled condition in the present campaign.

For trajectory *j*, containing $K_{j}$ evaluation instants, the reported gravity error is the time-averaged squared Euclidean full-vector error

(56) $$E_{g}^{\left(j\right)}=\frac{1}{K_{j}}\sum_{k=1}^{K_{j}}{∥g_{j,k}^{b}-{\hat{g}}_{j,k}^{b}∥}^{2}.$$

Because the gravity vector is expressed in $m/s^{2}$, $E_{g}^{\left(j\right)}$ has units of $m^{2}/s^{4}$. “Mean MSE” and “Maximum MSE” in Table 7 denote, respectively, the mean and maximum of $E_{g}^{\left(j\right)}$ over the trajectories in each scenario; they are not attitude-angle errors.

Table 7 summarizes the gravity-vector reconstruction error obtained for the different evaluation scenarios.

All 500 trajectories completed with a bounded gravity-vector estimate. The mean error increases monotonically from $2.17\times 10^{-3}\;m^{2}/s^{4}$ ($0.047\;m/s^{2}$ RMSE) under nominal conditions to $6.69\times 10^{-3}\;m^{2}/s^{4}$ ($0.082\;m/s^{2}$ RMSE) under combined disturbances, with an overall mean of $4.13\times 10^{-3}\;m^{2}/s^{4}$ ($0.064\;m/s^{2}$ RMSE). These values quantify body-frame gravity-vector reconstruction using the navigation-grade sensor suite of Table 4; attitude accuracy is a separate downstream metric.

**Figure 11:**
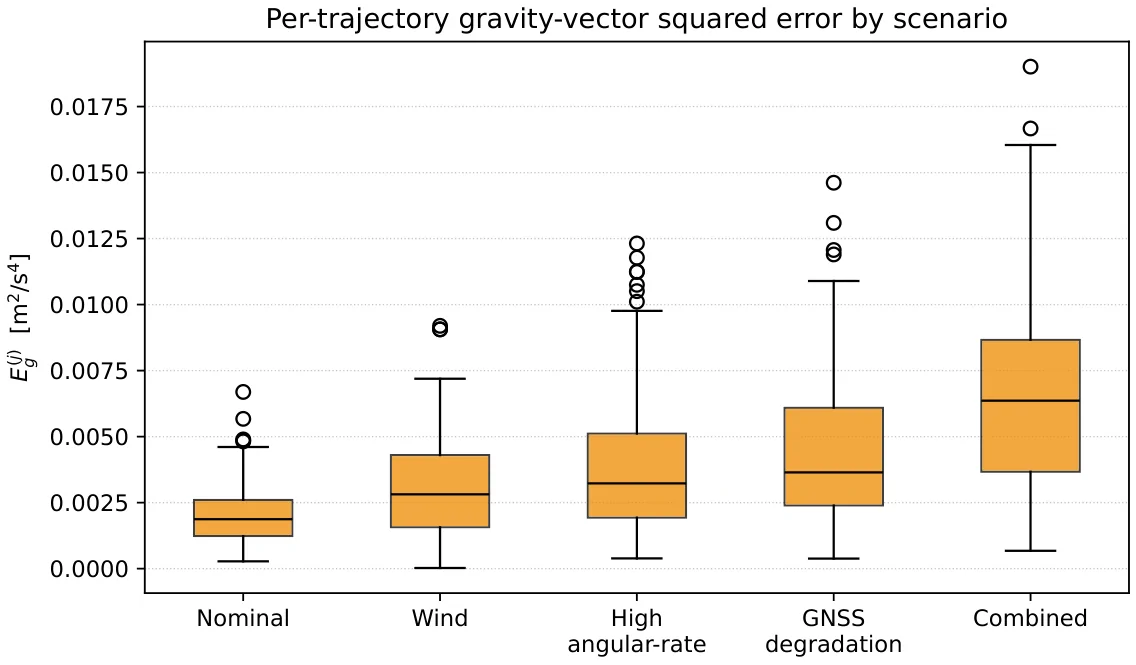
Distribution of the per-trajectory gravity-vector squared error $E_{g}^{\left(j\right)}$ by scenario (box: interquartile range; whiskers: 1.5 × IQR; line: median), over the trajectories in each scenario.

Figure 11 shows the full per-trajectory distribution of $E_{g}^{\left(j\right)}$ for each scenario, complementing the mean and maximum summary of Table 7.

The distributions widen and their upper tails lengthen from nominal to combined disturbances, consistent with the mean/maximum trend of Table 7; no scenario shows a bimodal or divergent tail, supporting the bounded, non-divergent operation reported below.

As discussed in Section 4, the reconstructed vector is incorporated into attitude determination together with GNSS-derived observations. The closed-loop runs show non-divergent operation of this coupling in all considered trajectories. The directly preceding two-network study reports the complementary attitude-restoration comparison with an RTK reference [50].

### 6.5. LOS Reconstruction Results

The third neural module focuses on line-of-sight (LOS) estimation, which constitutes one of the most critical quantities for terminal guidance. Although GNSS and inertial measurements provide accurate navigation information during most of the trajectory, LOS estimation becomes increasingly sensitive to navigation errors as the relative distance between the rocket and the target decreases.

The LOS-correction network was evaluated in the same 500 closed-loop trajectories used for gravity reconstruction. Consequently, the campaign covers nominal flight, wind, high-angular-rate maneuvers, GNSS degradation, and combined disturbances under a common set of operating conditions.

For trajectory *j*, the true unit LOS from the six-degree-of-freedom truth model is used only to calculate the time-averaged squared full-vector error

(57) $$E_{L}^{\left(j\right)}=\frac{1}{K_{j}}\sum_{k=1}^{K_{j}}{∥L_{\mathrm{true},j,k}^{n}-{\hat{L}}_{j,k}^{n}∥}^{2}.$$

Because both vectors are normalized, $E_{L}^{\left(j\right)}$ is dimensionless. For a fixed angular separation, the squared chordal error satisfies $∥L_{\mathrm{true}}-\hat{L}{∥}^{2}=2\left(1-\cos\Delta \psi \right)$. Because $E_{L}^{\left(j\right)}$ is already averaged over time, the angle obtained from it is an RMS-equivalent angular error rather than the arithmetic mean angular separation. We therefore define

(58) $$\Delta \psi_{\mathrm{eq}}^{\left(j\right)}=\arccos\;\left(1-\frac{E_{L}^{\left(j\right)}}{2}\right),$$

and report $\Delta \psi_{\mathrm{eq}}$ in degrees over both the complete trajectory and the SAL-active terminal window. The mean and maximum columns in Table 8 aggregate this trajectory-level metric.

The $\Delta \psi_{\mathrm{eq}}$ values in Table 8 are obtained by applying the closed-form chordal-error transformation to the reported mean squared unit-vector error. They are therefore RMS-equivalent angular errors, not arithmetic mean angular separations or directly aggregated epochwise angular RMSE values. For the observed error range ($E_{L}≲2.6\times 10^{-3}$, corresponding to angles below $3^{\circ }$), the small-angle relation $2\left(1-\cos\Delta \psi \right)≃\Delta \psi^{2}$ supports this physically interpretable approximation.

Table 8 summarizes the LOS-reconstruction error obtained for the different evaluation scenarios.

The residual LOS estimator remained numerically stable in all 500 trajectories. Its mean error increases from $8.74\times 10^{-4}$ under nominal conditions to $2.61\times 10^{-3}$ under combined disturbances, with an overall mean of $1.59\times 10^{-3}$. The corresponding RMS-equivalent angular error is 2.3° over the complete trajectory. Isolated to the SAL-active terminal window, it is larger (2.0° to 3.8° across scenarios and 2.8° overall), confirming that the full-trajectory figure understates the error during the phase that most directly affects terminal guidance. The SAL-active-window result should therefore be used when assessing terminal LOS adequacy.

**Figure 12:**
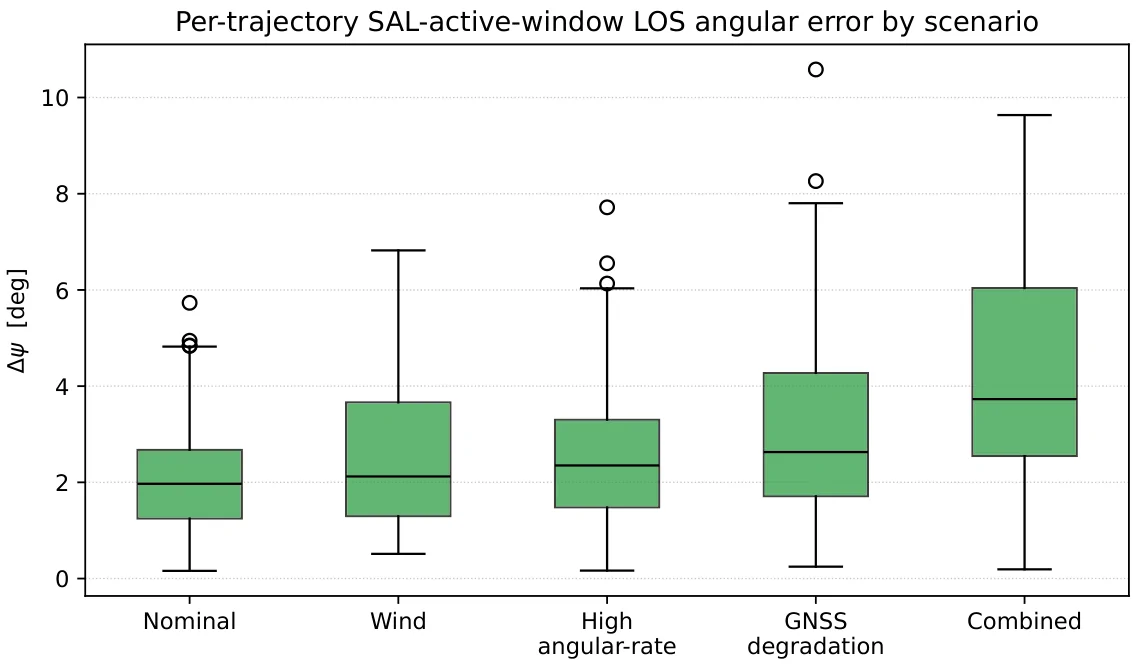
Distribution of the per-trajectory SAL-active-window RMS-equivalent LOS angular error $\Delta \psi_{\mathrm{eq}}$ (degrees) by scenario (box: interquartile range; whiskers: 1.5 × IQR; line: median).

Figure 12 shows the full per-trajectory distribution of the SAL-active-window angular error by scenario.

All trajectory sets were generated using the nonlinear six-degree-of-freedom rocket model described in Section 3, ensuring consistency between the neural-network training environment and the subsequent guidance and control simulations.

### 6.6. Hybrid Fusion Fallback Statistics

Section Model-Based Navigation Filter defined the numerical thresholds used to accept or reject each neural output. Table 9 reports, for the gravity and LOS modules, the percentage of closed-loop updates in which the neural output was accepted versus rejected (falling back to the model-based filter of Section Model-Based Navigation Filter or to the uncorrected geometric LOS, respectively). The corresponding ambiguity-module statistics are the fix and rejection rates already reported in Table 6 and are not repeated here.

For each scenario, the gravity-module acceptance and rejection percentages in Table 9 are computed over all eligible IMU-update epochs during the complete flight, whereas the LOS-module percentages are computed only over SAL-available epochs in the terminal window. Epochs outside the SAL-active window are excluded from the LOS denominator because use of the geometric LOS there is nominal operation rather than fallback. The final row is reported as the unweighted arithmetic mean of the five scenario-level percentages rather than as a pooled rate.

Rejection rates increase monotonically from nominal to combined disturbances for both modules, tracking the same disturbance ordering observed in Table 6, Table 7 and Table 8. In every rejection, the fallback value supplied by the filter of Section Model-Based Navigation Filter (or the uncorrected geometric LOS) is used in place of the neural output, so that no inadmissible neural output reaches the guidance law.

### 6.7. Terminal Guidance Performance: Miss Distance and CEP50

Table 10 reports the terminal miss distance for the complete three-module architecture and for the model-based baseline of Section 6.2, computed from the simulated impact point of each of the 500 evaluation trajectories relative to the designated target.

Overall CEP50 is defined as the median radial miss distance of the pooled 500-trajectory sample for each configuration. Because the median is nonlinear, this pooled statistic need not equal the arithmetic mean of the five scenario-specific CEP50 values. The same pooled-median definition is used in Table 10 and Table 11, the Abstract, the Discussion, and the Conclusions. Under this definition, CEP50 decreases from 0.49 m for the model-based baseline to 0.35 m for the three-module architecture, corresponding to a relative reduction of 28.6% (approximately 29%). Uncertainty was quantified using a paired bias-corrected-and-accelerated (BCa) bootstrap of the same 500 matched trials, computed directly from the original 500 matched baseline/full-architecture trajectory pairs over 10,000 bootstrap replicates. In each replicate, the same 500 trajectory indices were resampled with replacement as matched pairs and applied identically to both configurations, preserving the pairing, CEP50 was recomputed separately for the baseline and complete architecture, and the absolute reduction $\Delta \mathrm{CEP}50={\mathrm{CEP}50}_{baseline}-{\mathrm{CEP}50}_{full}$ was recorded. The observed absolute reduction is 0.140 m, with a paired-bootstrap 95% CI of [0.118,0.156] m, reported as the bias-corrected-and-accelerated (BCa) interval rather than a percentile interval.

The three-module architecture reduces overall CEP50 from 0.49 m (baseline) to 0.35 m, a relative reduction of approximately 29%, with the largest absolute reduction under combined disturbances (0.72 m to 0.53 m). These sub-meter values reflect the navigation-grade sensor suite of Table 4, and in particular, the precision SAL seeker used for terminal LOS correction. As with all other reported metrics, this is computed from the same closed-loop propagation: the impact point reflects the accepted (neural or fallback) navigation and LOS quantities as they influenced guidance and control throughout each trajectory.

### 6.8. Ablation Study

To isolate the contribution of each neural module, the closed-loop campaign was repeated with all eight combinations of the three modules enabled or disabled. Each configuration used the same 500 sets of initial conditions, disturbance realizations, sensor-noise sequences, and random seeds; the resulting propagated trajectories were allowed to differ because the estimator outputs altered the closed-loop guidance commands. All other GNC, sensor, and actuator settings were held fixed. Table 11 reports CEP50 for each configuration, together with the gravity and LOS mean squared errors where the corresponding module is enabled.

Using the pooled-median definition of Section 6.7, CEP50 decreases monotonically as neural modules are added, from 0.49 m for the fully model-based baseline to 0.35 m for the complete three-module architecture. The LOS-correction network yields the largest single-module improvement (0.49→0.42 m), consistent with its direct role in the guidance law of Equation (48); the ambiguity and gravity modules contribute smaller individual reductions, and no pairwise combination outperforms the full configuration. Table 10 and Table 11 use the same aggregation rule and therefore report identical baseline and full-configuration CEP50 values.

### 6.9. Integrated Evaluation Summary

Table 12 summarizes the role and directly reported evidence for each component of the integrated architecture.

The preceding studies are used only to identify methodological continuity. Neural LOS hybridization was examined as a specialized function in [48]; neural ambiguity candidate generation was evaluated in [49]; and ambiguity, gravity, and LOS-related onboard estimation were considered without the present matched three-module ablation in [50]. Because those studies used different campaigns, configurations, and metrics, their numerical results are not treated as benchmarks here. The relevant performance comparison for the present architecture is instead the matched baseline and eight-configuration ablation reported in Section 6.2, Section 6.3, Section 6.4, Section 6.5, Section 6.6, Section 6.7 and Section 6.8.

## 7. Discussion

The three neural modules operated concurrently within the propagated closed-loop simulation, with their outputs affecting the subsequent vehicle trajectory. Under combined disturbances, the mean gravity-vector and LOS errors increased by factors of 3.08 and 2.99, respectively, relative to nominal conditions. Despite this degradation, all errors remained finite and none of the 500 evaluated trajectories exhibited numerical divergence, supporting the feasibility of the integrated architecture over the tested operating conditions.

The matched baseline and ablation results clarify the mechanism of the system-level improvement. The LOS module produces the largest single-module reduction in CEP50 because its corrected angular estimate enters the proportional-navigation law directly. Ambiguity support and gravity reconstruction produce smaller individual changes, but they improve the attitude and navigation quantities used to transform and propagate LOS information. Their joint contribution is therefore complementary rather than redundant. The larger separation between the complete architecture and the baseline under GNSS degradation and combined disturbances is consistent with the intended role of the neural modules as auxiliary estimators when the conventional information sources are least reliable.

These results should nevertheless be interpreted as simulation-domain evidence. Training and evaluation use the same physical simulator family and a navigation-grade IMU/GNSS/SAL suite; consequently, the reported margins cannot yet be generalized to lower-grade sensors, unmodeled disturbances, processor timing constraints, or flight hardware. The acceptance and fallback logic limits the direct propagation of inadmissible neural outputs, but it does not establish formal closed-loop stability or external-domain robustness. Hardware-in-the-loop testing, sensitivity to sensor grade, and evaluation with independently generated data therefore remain necessary before operational conclusions can be drawn.

The three networks contain 32,022 trainable parameters in total (Section 4.1). Their forward-pass latency has not been profiled on flight-representative embedded hardware: the computational environment reported in Section 4.1 is the desktop workstation used for training and dataset generation. Consequently, no claim of real-time onboard execution is made at this stage; target-processor timing and hardware-in-the-loop validation remain necessary before suitability for flight deployment can be established.

The paired-bootstrap interval reported in Section 6.7 excludes zero, supporting the interpretation that the observed 0.140 m CEP50 reduction is a stable feature of the matched simulation comparison rather than an artifact of the particular 500-trajectory sample. This inference remains conditional on the modeled scenarios and sensor assumptions and does not establish external-domain or flight-test performance.

## 8. Conclusions

This paper has presented the simultaneous closed-loop integration of three specialized neural estimators within a model-based rocket GNC architecture. The modules generate GNSS ambiguity candidates, reconstruct the gravity vector in body axes, and estimate a residual correction to the GNSS/IMU-derived LOS using SAL and kinematic information. Their outputs remain embedded in the physical navigation, guidance, control-allocation, actuator, and six-degree-of-freedom vehicle chain rather than replacing that chain with an end-to-end neural controller.

The specific contribution is the concurrent representation of ambiguity, gravity, and residual LOS estimation as three explicit neural modules and their operation over a common five-scenario closed-loop campaign. The present work advances the specialized architectures summarized in the Introduction by (i) operating all three estimators simultaneously within one propagated closed loop, (ii) comparing their combined effect with a matched model-based configuration, and (iii) quantifying each module’s individual and joint contribution through an eight-configuration ablation study. Using the pooled-median aggregation adopted in Section 6.7, the complete architecture yields an approximately 29% reduction in terminal CEP50 (0.49→0.35 m) and a 1.5-percentage-point improvement in ambiguity fix success relative to the matched baseline.

At system level, the resulting architecture provides a more complete and resilient navigation chain: ambiguity support improves the availability of GNSS-derived attitude information, gravity reconstruction adds a complementary body-frame reference, and SAL-aided LOS correction reduces reliance on accumulated relative-position errors during terminal engagement. Physical validation and model-based fallback limit the propagation of inadmissible neural outputs into the guidance law while preserving the conventional GNC structure.

Across 500 evaluation trajectories, the gravity estimator achieved an overall mean trajectory squared-vector error of $4.13\times 10^{-3}\;m^{2}/s^{4}$, while the normalized LOS estimator achieved an overall mean squared unit-vector error of $1.59\times 10^{-3}$ (2.3° full-trajectory and 2.8° SAL-active-window RMS-equivalent angular errors). Under combined disturbances, the respective mean errors rose to 3.08 and 2.99 times their nominal values, but every trajectory completed without numerical divergence. Against the matched model-based baseline of Section 6.2, the complete architecture reduced overall terminal CEP50 from 0.49 m to 0.35 m and improved ambiguity fix success from 96.4% to 97.9% (Table 6 and Table 10), and the ablation study of Table 11 shows a near-additive, monotonically improving contribution as modules are added, with no pairwise combination outperforming the full configuration. The campaign therefore demonstrates simultaneous closed-loop operation of the three-module architecture over all tested scenarios, with a quantified, matched-baseline improvement rather than a demonstration of feasibility alone.

The evaluation uses a single simulation environment, built around the navigation-grade IMU/GNSS/SAL sensor suite of Table 4 and applied consistently to dataset generation, the matched baseline, and the ablation campaign. Both the matched baseline and the full ablation campaign (Section 6.2, Section 6.3, Section 6.4, Section 6.5, Section 6.6, Section 6.7 and Section 6.8) were evaluated over the complete 500-trajectory evaluation set; hardware-in-the-loop timing and flight-test evidence remain outside the present scope. Although complete evaluation trajectories and random seeds are separated from model-development data, external-domain generalization remains to be assessed.

Future work should therefore prioritize external-domain testing, sensitivity of the reported margins to lower-grade sensor suites, inference time on the target processor, and hardware-in-the-loop validation. The present campaign establishes the integrated closed-loop configuration and its matched-baseline, ablation-based evidence on which those system-level comparisons can be performed.

## Figures and Tables

**Table 1:** Functional progression from the directly related previous architectures to the present work.

Study	Sensor/Estimation Configuration	Principal Contribution	Reported Evidence
[18]	Model-based GNSS/IMU/SAL	Classical multisensor hybridization for rocket guidance	Closed-loop guidance and impact dispersion
[48]	Neural GNSS/IMU/SAL LOS hybridization	Neural prediction of terminal LOS information from navigation and quadrant-detector signals	Closed-loop LOS and impact CEP
[49]	Neural GNSS ambiguity estimation	Learning-based carrier-phase ambiguity processing for multi-antenna attitude determination	Ambiguity and attitude-estimation metrics
[50]	Neural ambiguity and gravity estimators, supplemented by SAL	Two learned vector sources embedded in an attitude-restoration chain	Monte Carlo angular restitution and RTK reference
Present work	Neural ambiguity, gravity, and residual LOS estimators	Simultaneous three-module integration in a common GNSS/IMU/SAL GNC loop	Module-level errors in a shared closed-loop disturbance campaign

**Table 2:** Trajectory-level partition used for neural-network development and closed-loop evaluation. The 500 evaluation trajectories are independent of all development subsets.

Subset	Trajectories	Share of Development Set	Purpose
Training	2800	70%	Parameter estimation
Validation	600	15%	Early stopping and model selection
Test	600	15%	Held-out network assessment
Closed-loop evaluation	500	Independent	Baseline, ablation, and scenario evaluation

**Table 3:** Network hyperparameters used for the three neural estimators.

Parameter	Value
Hidden layers	3
Neurons per layer	64
Activation function	ReLU (rectified linear unit)
Optimizer	Adam
Batch size	128
Initial learning rate	$10^{-3}$
Loss function	MSE (mean squared error)
Epochs	200

**Table 4:** Simulation and sensor parameters used throughout dataset generation and closed-loop evaluation.

Parameter	Value
Accelerometer bias, 1σ ($b_{a}$)	$3\times 10^{-4}\;g$
Accelerometer noise density ($n_{a}$)	$25\;\mu g/\sqrt{\mathrm{Hz}}$
Gyroscope bias, 1σ ($b_{g}$)	$0.5^{\circ }/h$
Gyroscope noise density ($n_{g}$)	$0.003^{\circ }/s/\sqrt{\mathrm{Hz}}$
Pseudorange noise, 1σ (nominal)	0.1 m
Carrier-phase noise, 1σ (nominal)	1 mm
GNSS degradation: pseudorange noise increase	×3 (0.3 m)
GNSS degradation: carrier-phase noise increase	×2.5 (2.5 mm)
GNSS degradation: visible satellites	8–10 (nominal) → 4–5 (degraded)
GNSS degradation: outage duration	2–5 s (uniformly sampled)
SAL detector angular noise, 1σ	0.012°
SAL boresight misalignment, 1σ	0.004°
Navigation constant, *N* (proportional-navigation law)	4
Maximum canard deflection, $\delta_{max}$	$18^{\circ }$
Maximum canard deflection rate, ${\dot{\delta }}_{max}$	$150^{\circ }/s$
IMU update rate	200 Hz
GNSS update rate (position/velocity/carrier phase)	10 Hz
SAL update rate	100 Hz

**Table 5:** Evaluation scenario definitions. The five scenarios together comprise the full 500-trajectory six-degree-of-freedom evaluation campaign.

Scenario	Wind (±15 m/s)	Angular Rate (Up to $200^{\circ }$/s)	GNSS Degradation	Aerodynamic Uncertainty
Nominal	No	No	No	No
Wind disturbances	Yes	No	No	No
High angular-rate maneuvers	No	Yes	No	No
GNSS degradation periods	No	No	Yes (Table 4)	No
Combined disturbances	Yes	Yes	Yes (Table 4)	Yes

**Table 6:** Ambiguity fix success, false-fix, and rejection rates per scenario for the neural candidate-generation chain and for the LAMBDA-only baseline.

	Neural Candidate + Validation			LAMBDA-Only Baseline		
Scenario	Fix (%)	False Fix (%)	Reject (%)	Fix (%)	False Fix (%)	Reject (%)
Nominal	99.3	0.1	0.6	99.0	0.2	0.9
Wind disturbances	99.0	0.2	0.9	98.3	0.3	1.5
High angular-rate	98.6	0.2	1.2	97.5	0.4	2.2
GNSS degradation	97.1	0.4	2.5	95.0	0.8	4.2
Combined disturbances	95.3	0.6	4.1	92.1	1.2	6.7
Overall (scenario mean)	97.9	0.3	1.9	96.4	0.6	3.1

**Table 7:** Closed-loop gravity-vector reconstruction errors over the six-degree-of-freedom evaluation trajectories. MSE units are $m^{2}/s^{4}$.

Scenario	Mean MSE	Max. MSE
Nominal flight conditions	$2.17\times 10^{-3}$	$6.90\times 10^{-3}$
Wind disturbances	$3.10\times 10^{-3}$	$9.34\times 10^{-3}$
High angular-rate maneuvers	$4.09\times 10^{-3}$	$1.16\times 10^{-2}$
GNSS degradation periods	$4.59\times 10^{-3}$	$1.35\times 10^{-2}$
Combined disturbances	$6.69\times 10^{-3}$	$1.96\times 10^{-2}$
Overall evaluation set (500 traj.)	$4.13\times 10^{-3}$	$1.96\times 10^{-2}$

**Table 8:** Closed-loop LOS reconstruction errors over the six-degree-of-freedom evaluation trajectories. The squared unit-vector error is dimensionless; $\Delta \psi_{\mathrm{eq}}$ is the RMS-equivalent angular error in degrees.

Scenario	Mean MSE	Max. MSE	$\Delta \psi_{\mathrm{eq}}$, Full Traj. (deg)	$\Delta \psi_{\mathrm{eq}}$, SAL-Active Window (deg)
Nominal flight conditions	$8.74\times 10^{-4}$	$2.96\times 10^{-3}$	1.7	2.0
Wind disturbances	$1.17\times 10^{-3}$	$3.84\times 10^{-3}$	2.0	2.4
High angular-rate maneuvers	$1.43\times 10^{-3}$	$4.67\times 10^{-3}$	2.2	2.7
GNSS degradation periods	$1.85\times 10^{-3}$	$5.93\times 10^{-3}$	2.5	3.1
Combined disturbances	$2.61\times 10^{-3}$	$7.84\times 10^{-3}$	2.9	3.8
Overall evaluation set (500 traj.)	$1.59\times 10^{-3}$	$7.84\times 10^{-3}$	2.3	2.8

**Table 9:** Acceptance and rejection (fallback) rates of the gravity-vector and LOS-correction neural outputs, by scenario, under the numerical thresholds of Section Model-Based Navigation Filter.

	Gravity Module		LOS Module	
Scenario	Accepted (%)	Rejected (%)	Accepted (%)	Rejected (%)
Nominal	99.7	0.3	99.9	0.1
Wind disturbances	99.5	0.5	99.8	0.2
High angular-rate	99.2	0.8	99.8	0.2
GNSS degradation	98.6	1.4	99.6	0.4
Combined disturbances	97.7	2.3	99.2	0.8
Overall (scenario mean)	98.9	1.1	99.7	0.3

**Table 10:** Terminal miss distance (mean, CEP50, and maximum, in meters) by scenario, for the three-module neural architecture and the model-based baseline.

	Three-Module Architecture			Model-Based Baseline		
Scenario	Mean (m)	CEP50 (m)	Max (m)	Mean (m)	CEP50 (m)	Max (m)
Nominal	0.26	0.22	0.68	0.30	0.26	0.78
Wind disturbances	0.34	0.29	0.90	0.40	0.35	1.10
High angular-rate	0.38	0.32	1.03	0.47	0.40	1.25
GNSS degradation	0.47	0.41	1.33	0.63	0.54	1.73
Combined disturbances	0.62	0.53	1.70	0.86	0.72	2.28
Overall (500 traj.)	0.41	0.35	1.70	0.53	0.49	2.28

**Table 11:** Ablation study: terminal-guidance CEP50 and module-level mean squared errors for every combination of the three neural modules, evaluated over the complete 500-trajectory evaluation campaign. A checkmark indicates that the corresponding neural module is enabled.

Configuration	Amb.-NN	Grav.-NN	LOS-NN	CEP50 (m)	Gravity MSE	LOS MSE
Baseline (none)	—	—	—	0.49	—	—
Ambiguity-NN only	✓			0.46	—	—
Gravity-NN only		✓		0.44	$4.36\times 10^{-3}$	—
LOS-NN only			✓	0.42	—	$1.71\times 10^{-3}$
Ambiguity + Gravity	✓	✓		0.40	$4.20\times 10^{-3}$	—
Ambiguity + LOS	✓		✓	0.38	—	$1.64\times 10^{-3}$
Gravity + LOS		✓	✓	0.37	$4.13\times 10^{-3}$	$1.61\times 10^{-3}$
All three (full)	✓	✓	✓	0.35	$4.13\times 10^{-3}$	$1.59\times 10^{-3}$

**Table 12:** Role and evaluation evidence for the integrated modules.

Module	Role in the Architecture	Present Evidence
GNSS ambiguity estimator	Candidate generation for carrier-phase processing	Regression-error distribution; fix/false-fix/rejection rates vs. LAMBDA baseline (Table 6)
Gravity reconstruction network	Additional body-frame vector for attitude restitution	Scenario-wise squared vector error and RMSE; acceptance/fallback rate (Table 7 and Table 9)
LOS correction network	Residual GNSS/IMU/SAL LOS correction	Scenario-wise squared unit-vector error and angular error, full-trajectory and SAL-active window; acceptance/fallback rate (Table 8 and Table 9)
Complete GNC loop	Propagation through guidance, allocation, actuation, and vehicle dynamics	500 closed-loop trajectories without numerical divergence; terminal miss-distance/CEP50 vs. model-based baseline (Table 10); eight-configuration ablation study (Table 11)

## Data Availability

Data and simulation models will be made available upon reasonable request, subject to institutional and project restrictions.

## Abbreviations

The following abbreviations are used in this manuscript:

GNSS	Global Navigation Satellite System
IMU	Inertial Measurement Unit
SAL	Semi-Active Laser
GNC	Guidance, Navigation, and Control
LOS	Line of Sight
NED	North-East-Down
INS	Inertial Navigation System
RTK	Real-Time Kinematic
CEP	Circular Error Probable
DD	Double-Difference
LAMBDA	Least-Squares Ambiguity Decorrelation Adjustment
EKF	Extended Kalman Filter
MSE	Mean Squared Error
RMSE	Root Mean Squared Error
NN	Neural Network
ReLU	Rectified Linear Unit
DOF	Degree(s) of Freedom
PPP	Precise Point Positioning

## Nomenclature

The following symbols are used in this manuscript:

*Reference frames and kinematics*	
$F_{n}$	Navigation (NED) reference frame
$F_{b}$	Body reference frame
$X_{N},Y_{E},Z_{D}$	NED frame axes
$X_{B},Y_{B},Z_{B}$	Body frame axes
$C_{b}^{n}$	Direction cosine matrix, body to navigation frame
$v^{n},v^{b}$	Rocket velocity, navigation/body frame
$r_{rel}^{n}$	Relative position vector, rocket to target
$r_{T}^{n},r_{R}^{n}$	Target/rocket position, navigation frame
$L^{n},L^{b}$	LOS unit vector, navigation/body frame
*Six-degree-of-freedom rigid-body dynamics*	
*m*	Vehicle mass
$F_{T}^{n}$	Thrust force
$F_{A}^{n},F_{A}^{b}$	Aerodynamic force
$F_{C}^{n},F_{C}^{b}$	Control (canard) force
$g^{n},g^{b}$	Gravity vector, navigation/body frame
*g*	Local gravitational-acceleration magnitude
J	Inertia tensor
$\omega^{b}$	Body angular-rate vector
$M_{A}^{b}$	Aerodynamic moment
$M_{C}^{b}$	Control (canard) moment
$q={\left[q_{0},q_{v}^{T}\right]}^{T}$	Attitude quaternion
ρ	Local air density
*d*	Rocket reference diameter
$C_{X},C_{Y},C_{Z}$	Aerodynamic force coefficients
$C_{l},C_{m},C_{n}$	Aerodynamic moment coefficients
$\delta_{h},\delta_{v}$	Horizontal/vertical canard deflection
$C_{X_{\delta_{h}}},C_{Y_{\delta_{h}}},C_{Z_{\delta_{h}}}$	Horizontal-canard control force derivatives
$C_{X_{\delta_{v}}},C_{Y_{\delta_{v}}},C_{Z_{\delta_{v}}}$	Vertical-canard control force derivatives
$C_{l_{\delta_{h}}},C_{m_{\delta_{h}}},C_{n_{\delta_{h}}}$	Horizontal-canard control moment derivatives
$C_{l_{\delta_{v}}},C_{m_{\delta_{v}}},C_{n_{\delta_{v}}}$	Vertical-canard control moment derivatives
*IMU, GNSS, and SAL sensor models*	
${\tilde{f}}^{b}$	Measured specific force
$f^{b}$	True (ideal) specific force
$b_{a},n_{a}$	Accelerometer bias/measurement noise
${\tilde{\omega }}^{b}$	Measured angular-rate vector
$b_{g},n_{g}$	Gyroscope bias/measurement noise
$\rho_{i}$	Pseudorange measurement, satellite *i*
$R_{i}$	Geometric range
*c*	Speed of light
$\delta t_{r},\delta t_{i}$	Receiver/satellite clock offset
$I_{i},T_{i}$	Ionospheric/tropospheric delay
$\epsilon_{\rho ,i}$	Pseudorange noise and residual errors
$\Phi_{i}$	Carrier-phase measurement
$N_{i}$	Integer ambiguity (cycles)
λ	Carrier wavelength
$\epsilon_{\Phi ,i}$	Carrier-phase residual error
$\Phi_{DD,j}$	Double-difference carrier-phase measurement
$R_{DD,j}$	Differenced geometric range
$T_{DD,j},I_{DD,j}$	Differenced atmospheric-delay terms
$N_{DD,j}$	Double-difference integer ambiguity
$\varepsilon_{\Phi ,DD,j}$	Double-difference carrier-phase residual error
$N_{DD}$	Double-difference ambiguity vector
A,B,C,D	SAL quadrant-detector outputs
u,v	Normalized detector spot coordinates
$\theta_{LOS},\psi_{LOS}$	Seeker LOS angles
$f_{u},f_{v}$	Detector calibration functions
x	Full sensor-fusion state vector
*Ambiguity-support network*	
$z_{GNSS}$	Ambiguity-network input feature vector
$\rho_{DD}$	Differenced pseudorange features
$\Phi_{DD}$	Differenced carrier-phase features
$d_{sat}$	Satellite-geometry indicators
$s_{q}$	Signal-quality metrics
$f_{N}$	Ambiguity-network mapping
${\hat{N}}_{DD}$	Estimated double-difference ambiguity candidates
*J*	Training loss function
*M*	Number of training samples
*Gravity-reconstruction network*	
$z_{g}$	Gravity-network input feature vector
${\hat{g}}^{b}$	Estimated gravity vector, body frame
$f_{g}$	Gravity-network mapping
*LOS-correction network*	
$L_{GI}^{n}$	GNSS/IMU geometric LOS estimate
$L_{SAL}^{b},L_{SAL}^{n}$	SAL LOS estimate, body/navigation frame
$z_{L}$	LOS-network input feature vector
$\Delta L^{n}$	Estimated LOS residual
$f_{L}$	LOS-network mapping
${\hat{L}}^{n}$	Corrected (reconstructed) LOS estimate
$\epsilon_{L}$	LOS normalization tolerance
*Hybrid fusion and model-based (EKF) filter*	
$x_{F}$	Hybrid fusion vector
δx	15-element EKF error-state vector
δθ	Attitude (small-angle) error
$\delta v^{n}$	Velocity error
$\delta r^{n}$	Position error
$\delta b_{a},\delta b_{g}$	Accelerometer-/gyroscope-bias error
$P_{k}^{-},P_{k}^{+}$	A priori/a posteriori error covariance
$\Phi_{k-1}$	Discrete-time state-transition matrix
$Q_{k-1}$	Process-noise covariance
$z_{GNSS,k}$	GNSS measurement, EKF update
$H_{GNSS}$	GNSS measurement selection matrix
$n_{GNSS,k}$	GNSS measurement noise
$z_{g,k}$	Gravity-constraint measurement, EKF update
${\hat{C}}_{b,k}^{n-}$	A priori attitude estimate
$n_{g,k}$	Gravity measurement noise
$K_{k}$	Kalman gain
$R_{k}$	Measurement-noise covariance
I	Identity matrix
*Guidance and control*	
$V_{c}$	Closing velocity
$v_{rel}^{n}$	Relative velocity vector
$v_{T}^{n},v_{R}^{n}$	Target/rocket velocity
*N*	Navigation constant
$a_{c}^{n},a_{c}^{b}$	Commanded acceleration, navigation/body frame
${\dot{\hat{L}}}^{n}$	Estimated LOS rate
$\hat{R}$	Estimated target range
${\hat{v}}_{rel}^{n}$	Estimated relative velocity
$a_{lat}^{b}$	Desired lateral acceleration
$a_{Y,c},a_{Z,c}$	Commanded lateral acceleration components
$\delta_{c}$	Canard command vector ${\left[\delta_{h}\;\delta_{v}\right]}^{T}$
B	Control-effectiveness matrix
$B^{†}$	Moore–Penrose pseudoinverse of B
$a_{0}^{b}$	Lateral acceleration at zero canard deflection
$\delta_{max}$	Maximum canard deflection
${\dot{\delta }}_{max}$	Maximum canard deflection rate
*Evaluation metrics*	
*j*	Trajectory index
$K_{j}$	Number of evaluation instants in trajectory *j*
$E_{g}^{\left(j\right)}$	Trajectory-averaged squared gravity-vector error
$E_{L}^{\left(j\right)}$	Trajectory-averaged squared LOS unit-vector error
Δψ	LOS angular error
